# Integrative cardiovascular dose–response to graded lower‐body negative pressure

**DOI:** 10.1113/EP092483

**Published:** 2025-05-14

**Authors:** Richard S. Whittle, Nathan Keller, Eric A. Hall, Safiyya Patanam, Bonnie J. Dunbar, Ana Diaz‐Artiles

**Affiliations:** ^1^ Department of Mechanical and Aerospace Engineering University of California Davis California USA; ^2^ Department of Aerospace Engineering Texas A&M University College Station Texas USA; ^3^ Department of Kinesiology and Sport Management Texas A&M University College Station Texas USA; ^4^ School of Engineering Medicine (EnMed) Texas A&M University College Station Texas USA; ^5^ Department of Biomedical Engineering Texas A&M University College Station Texas USA

**Keywords:** Bayesian models, cardiovascular deconditioning, dose–response, lower‐body negative pressure, spaceflight countermeasures

## Abstract

Lower‐body negative pressure (LBNP) has been posited as a potential spaceflight countermeasure to counteract the physiological deconditioning related to fluid shifts in microgravity. However, open questions remain regarding the magnitude of LBNP that should be applied. We systematically characterized the cardiovascular effects of LBNP and quantified the effect size of varied LBNP doses across different parts of the cardiovascular system. Twenty‐four subjects (12 male and 12 female) were exposed to graded LBNP, increasing from 0 to −50 mmHg in 10 mmHg increments, in both supine (0°) and 15° head‐down tilt postures. At each pressure level, subjects first underwent a 6 min stabilization period to reach a steady‐state cardiovascular response. We then assessed a wide range of variables, including those related to the systemic circulation, cardiovascular control, and haemodynamics of the eyes and neck. Building on the experimental data, dose–response curves were constructed using a Bayesian multivariate hierarchical modelling framework to quantify the effect size of every variable considered when subjected to LBNP. The methodology allows direct comparison of the variables and the underlying structural relationships between them. Furthermore, we demonstrated the potential for LBNP to reduce jugular venous flow stagnation, which is considered one of the major health risks during human spaceflight. The dose–response curves and effect sizes generated from this research effort establish the most comprehensive framework available to date that characterizes physiological responses to LBNP. These results directly inform the development of countermeasures to mitigate the negative effects of spaceflight, including cardiovascular deconditioning, spaceflight‐associated neuro‐ocular syndrome and venous thromboembolism events.

## Abstract (highlights)

- **What is the central question of this study?**
  This study evaluates lower‐body negative pressure (LBNP) as a countermeasure for spaceflight risks, including cardiovascular deconditioning, spaceflight‐associated neuro‐ocular syndrome, and venous thromboembolism events. The central question focuses on determining the LBNP dose required to mitigate these risks.
- **What is the main finding and its importance?**
  Using supine and 15° head‐down tilt positions, we generated LBNP dose–response curves to assess haemodynamics, autonomic, ocular, and neck responses across various LBNP levels. A Bayesian multivariate framework revealed significant inter‐variable relationships and effect size differences. Key findings highlight the potential of LBNP to reduce jugular venous flow stagnation during microgravity. This comprehensive analysis informs countermeasure development to address spaceflight‐induced physiological challenges.

## 1 INTRODUCTION

Future long‐duration exploration missions will require novel countermeasure protocols to mitigate the degrading effects of the microgravity environment. Specifically relevant to the cardiovascular system is the redistribution of fluids that occurs when entering microgravity conditions. In particular, three of the risks directly related to the fluid shift identified by the National Aeronautics and Space Administration (NASA) Human Research Program (Antonsen et al., 2022) are: (1) the risk of cardiovascular adaptations contributing to adverse mission performance and health outcomes (Hargens & Richardson, 2009); (2) the risk of spaceflight associated neuro‐ocular syndrome (SANS; Lee et al., 2018; Lee et al., 2020; Stenger et al., 2017); and (3) the concern of venous thromboembolism (VTE; Auñón‐Chancellor et al., 2020; Harris et al., 2022; Kim et al., 2021; Limper et al., 2021; Marshall‐Goebel et al., 2019; Simka et al., 2020; Zwart et al., 2022). Lower‐body negative pressure (LBNP) has a long spaceflight heritage since the Skylab Program in the 1970s (Johnson et al., 1975; Johnson et al., 1977), both for human physiology research and a countermeasure to prevent post‐flight orthostatic intolerance. Currently, Russian cosmonauts on the International Space Station (ISS) use the Chibis‐M suit, developed in 2012, as a countermeasure prior to landing (Campbell & Charles, 2015). The Chibis protocol, developed by the Institute for Biomedical Problems of the Russian Academy of Sciences (IBMP), is short, consisting of 2 min at −25 mmHg followed by 3 min at −35 mmHg (Gazenko et al., 1981). The primary effect of LBNP is to pool blood down towards the feet, reducing venous return and introducing central hypovolaemia (Goswami et al., 2019). Thus, LBNP is also used terrestrially to study the effects of acute hypovolaemia and haemorrhagic shock (Convertino, 2001; Hinojosa‐Laborde et al., 2014). Although LBNP does not restore hydrostatic gradients or affect tissue weight, this footward fluid shift could counteract the microgravity‐induced cephalad fluid shift. LBNP has been demonstrated to effectively reduce intraocular pressure (IOP; Greenwald et al., 2021), intracranial pressure (ICP; Petersen et al., 2019), and optic nerve sheath diameter (Marshall‐Goebel et al., 2017) in multiple ground‐based studies. In addition to its use as a countermeasure to prevent orthostatic intolerance, LBNP has also been posited as a potential countermeasure to mitigate cardiovascular degradation, SANS, and VTE events during long‐duration spaceflight (Petersen et al., 2019).

To develop successful LBNP protocols effectively, it is important to quantify fully the effects of different levels of negative pressure on different aspects of the cardiovascular system. This is limited not only to the systemic haemodynamics, but also a complete and comprehensive understanding of the haemodynamic and autonomic response. Additionally, and particularly important for SANS and VTE, it is also necessary to quantify the specific effects on the haemodynamics of the ocular system and of the head and neck. Finally, there is a large amount of individual variation between crewmembers in terms of both anthropometry (Whittle & Diaz‐Artiles, 2021) and LBNP tolerance (Lightfoot et al., 1991). Thus, it is also important to characterize any relationships between cardiovascular variables and easily measurable subject characteristics, such as age, height, and weight. As one example, Buckey et al. (2022) have previously identified an association between IOP changes in microgravity and body weight. This is particularly important as the profile of spaceflight participants broadens with the rise of commercial spaceflight (Hodkinson et al., 2017).

LBNP has been studied extensively in the literature (Campbell & Charles, 2015; Convertino et al., 2004; Franke et al., 2003; Goswami et al., 2009, 2008, 2019; Hinojosa‐Laborde et al., 2014; Hisdal et al., 2002; Johnson, 1979; Kelly et al., 2004; Khan et al., 2002; Lightfoot et al., 1991; Marshall‐Goebel et al., 2017; Petersen et al., 2019; Scherrer et al., 1988). Multiple studies have looked at the difference between males and females, with the majority of them noting a difference in orthostatic tolerance (Carter et al., 2015; Convertino, 1998; Franke et al., 2003; Frey & Hoffler, 1988; Monahan & Ray, 2004; Patterson et al., 2022; White et al., 1996). For example, Patterson et al. (2022) highlight the importance of sex as a factor in the cardiovascular response to LBNP. Fong et al. (2007) posited that the lower orthostatic tolerance observed in females (during centrifugation) was likely to be attributable to the combination of anthropometric factors, the vasoactive effects of sex hormones, and structural differences in cardiac anatomy. However, none of the studies mentioned has considered the same range of haemodynamic, autonomic, and head/neck measurements considered in the present study. Furthermore, there are some studies that examine tilt and LBNP as two separate interventions (e.g., Greenwald et al., 2021, Ogoh et al., 2022, Patterson et al., 2022). However, we could find only a few studies in which both tilt and LBNP are considered together, that is, LBNP during head‐up tilt (HUT) or head‐down tilt (HDT). In most of these cases, only a single value or a small range of LBNP is examined (Lawley et al., 2020; Macias et al., 2015; Patel et al., 2016). Finally, the Bayesian workflow that we introduce in this study is noteworthy, with no previous studies examining the network of associations between variables.

The aim of this study was to construct dose–response curves to quantify the acute response of the cardiovascular system across a range of LBNP levels. We aimed to encompass a wide range of systemic and autonomic parameters, in addition to variables related to the head and neck, in order to provide a holistic picture of the integrated response to LBNP, particularly as it relates to potential use cases as a spaceflight countermeasure. Although other studies have considered the acute response to LBNP across multiple cardiovascular variables, we intended to focus on the spaceflight application and answer the question, ‘How much LBNP is required to compensate for the changes induced by a cephalad fluid shift?’ in any given variable of interest. We also intended to quantify any sex‐dependent differences in LBNP response. Finally, in the process of analysing our data, we developed a novel workflow for the construction of dose–response curves using Bayesian multivariate analysis. This multivariate framework captured the relationships between all the measured variables, in addition to subject characteristics such as age, height, and weight. Such a methodology could be expanded beyond the cardiovascular system to encompass other organ systems. Together, these results lead to a greater understanding of LBNP as a potential spaceflight countermeasure, informing the development of future protocols.

## 2 MATERIALS AND METHODS

### 2.1 Subjects and study approval

Twenty‐four healthy, recreationally active subjects (12 male and 12 female) between 22 and 42 years of age were recruited from the Texas A&M University System to participate in the study. Subjects were matched for age and body mass index (BMI) between the male and female groups. Sample size and the number of pressure levels required were determined based on a power curve analysis of pilot data. Subject characteristics (mean ± SD) are presented in Table 1. Table 1 also gives the Bayes factor ($BF_{10}$), which is a Bayesian term that represents the strength of the evidence supporting differences between the male and female groups. Thus, the larger the $BF_{10}$, the more evidence to reject the null hypothesis that the standardized effect size of the difference in a characteristic between groups (d) is negligible [where negligible is defined by a region of practical equivalence (ROPE) of ±0.1; Kruschke (2018) suggests ±0.1 as a default value for a standardized parameter, equivalent to a negligible effect size according to Cohen (Cohen, 1988)], such that:

(1) $$H_{0}:-0.1<d<0.1$$

**TABLE 1:** Characteristics of the 24 recreationally active subjects (12 males and 12 females) who participated in the study.

Characteristic	Male	Female	$BF_{10}$
n	12	12	–
Age (years)	28.8 ± 5.4	28.1 ± 4.2	0.335
Height (cm)	178.9 ± 7.0	160.3 ± 7.8	50,800^c^
Weight (kg)	86.1 ± 18.9	62.9 ± 16.4	10.4^b^
BMI (kg/m^2^)	26.8 ± 5.2	24.5 ± 6.3	0.492
HR (beats/min)	73.8 ± 12.0	75.8 ± 11.5	0.341
MAP (mmHg)	106.8 ± 12.4	94.9 ± 5.4	7.81^a^

*Note*: Characteristics were recorded during the baseline session prior to testing sessions. Data are reported as the mean ± SD where appropriate. $BF_{10}$ represents the strength of the evidence supporting differences between the male and female groups.

Abbreviations: BMI, body mass index; HR, heart rate; MAP, mean arterial pressure.

^a^ Moderate evidence supporting differences between the male and female groups.

^b^ Strong evidence supporting differences between the male and female groups.

^c^ Extreme evidence supporting differences between the male and female groups.

Prior to participating in the study, subjects completed a questionnaire designed to identify any exclusion criteria, including current use of any cardiac, blood pressure, muscle relaxant, anticoagulant or stimulant medications, thyroid disease, chronic cardiovascular pathologies, extreme obesity, history of hypertension, or possible pregnancy. Each subject received written and verbal explanations of the study protocols and gave written informed consent to participate in the experiment. All procedures performed in the study were in accordance with the *1964 Helsinki Declaration* and its later amendments. The study protocol was approved by the Texas A&M Human Research Protection Program with Institutional Review Board number IRB2020‐0724F.

### 2.2 Experimental design and testing protocol

The experiment followed a counterbalanced, within‐subjects design, in which each participant experienced all pressure levels and postures. Subjects were exposed to progressively increasing LBNP from 0 to −50 mmHg in 10 mmHg increments, in two separate postures: 0° supine (face‐up) and 15° HDT supine. Each posture was tested on a separate day, with experimental sessions conducted within a 2 week period in a counterbalanced order.

During each experimental session (0° supine or 15° HDT), subjects were placed in an LBNP chamber (Technavance, Austin, TX, USA) at an initial pressure of 0 mmHg relative to atmospheric pressure. Continuous measurements of blood pressure and electrocardiography were recorded throughout the test. Subjects initially remained at rest for a period of 6 min to allow any haemodynamic transients to settle. After the rest period, an inert gas rebreathing device was used to collect discrete measurements of cardiac parameters. Following this, measurements of ocular tonometry, ultrasonography, and non‐invasive measurement of internal jugular venous pressure were also collected. This data collection sequence, including the initial 6 min rest period, lasted for ∼12 min per pressure level. The LBNP level was then increased (i.e., made more negative) by 10 mmHg and the entire data collection sequence repeated (i.e., a 6 min resting period followed by 6 min of discrete measurements for a total of 12 min). The protocol included six pressure levels: 0, −10, −20, −30, −40 and −50 mmHg, totalling ∼72 min per session. To control for potential circadian effects, all sessions were scheduled in the morning at approximately the same time. In addition, subjects were asked to refrain from drinking caffeine and exercising prior to each session.

Additionally, before the first experimental session (0° supine or 15° HDT, counterbalanced), baseline measurements were collected in a seated posture without LBNP. In this baseline session, subjects sat at rest for 6 min, before undergoing the same discrete measurements collected in the same order as in the main experimental sessions.

Testing was discontinued immediately if subjects experienced discomfort or presented physiological markers of presyncope, such as unrestrained rising heart rate (HR), falling blood pressure and/or profuse perspiration. In the 0° supine position, presyncope was reached in seven subjects at −40 mmHg (n=2, both female) and −50 mmHg (n=5, 4 females, 1 male). In the 15° HDT position, presyncope was reached in two subjects (both female) at −40 mmHg (n=1, one of them also reached presyncope at −40 mmHg whilst at 0° supine) and −50 mmHg (n=1, also experienced presyncope at −50 mmHg whilst in 0° supine). After the discontinued application of LBNP, no subjects experienced lasting symptoms. The remainder of the data for these subjects up to the point of discontinuation are included in the results. All other subjects completed the full protocol and experienced no adverse effects.

### 2.3 Dependent variables

Dependent variables included 11 haemodynamic metrics, four autonomic indices, and eight measures related to the head/neck/eyes. The haemodynamic measurements considered were: (1) heart rate (HR, in beats per minute); (2) stroke volume (SV, in millilitres); (3) cardiac output (CO, in litres per minute); (4) oxygen consumption (${\dot{V}}_{O_{2}}$, in litres per minute); (5) systolic blood pressure (SBP, in millimetres of mercury); (6) diastolic blood pressure (DBP, in millimetres of mercury); (7) rate–pressure product (RPP, in millimetres of mercury per minute), used as a metric for myocardial stress and energy consumption (Ansari et al., 2012); (8) myocardial oxygen supply:demand index (MO, calculated as the ratio of the diastolic pressure time interval to the systolic pressure time interval, DPTI/SPTI, no units) (Hoffman & Buckberg, 2014); and (9) total peripheral resistance (TPR, in millimetres of mercury multiplied by seconds per millilitre). Additionally, two body‐weight‐normalized indices were collected: (10) stroke index (SI, in millilitres per metre squared); and (11) cardiac index (CI, in litres per minute per metre squared).

Following the recommendations of the Task Force of the European Society of Cardiology and the North American Society of Pacing and Electrophysiology (TFESCNASPE, 1996), we collected four time‐domain autonomic indices. These measurements were: (1) the standard deviation of the NN intervals (SDNN, in milliseconds); (2) heart rate variability triangular index (HRVTi, no units); (3) the root mean square of direct differences of the NN interval (RMSDD, in milliseconds); and (4) baroreflex sensitivity (BRS, in milliseconds per millimetre of mercury). SDNN and HRVTi represent heart rate variability incorporating sympathetic and parasympathetic effects, RMSDD is closely correlated with parasympathetic activity (Berntson et al., 2005), and BRS represents a measure of total autonomic control via the arterial baroreflex (Eckberg & Sleight, 1992; Rovere et al., 2008; TFESCNASPE, 1996). BRS was measured via cross‐correlation between beat‐to‐beat SBP and delayed RR interval (a 5 s delay to account for sympathetically mediated reflexes) across a sliding 10 s window, according to procedures developed and validated by Westerhof et al. (2004).

In relationship to the head and neck, the following eight measurements were collected (or calculated): (1) IOP (in millimetres of mercury); (2) ocular perfusion pressure (OPP, in millimetres of mercury); (3) internal jugular vein cross‐sectional area (*A*_IJV_, in millimetres squared); (4) internal jugular vein pressure (IJVP, in millimetres of mercury); (5) internal jugular vein flow pattern (IJVF, grade; see Section 2.4); (6) common carotid artery cross‐sectional area (*A*_CCA_, in millimetres squared); (7) common carotid artery peak systolic velocity (PSV, in centimetres per second); and (8) common carotid artery end‐diastolic velocity (EDV, in centimetres per second). Head and neck measurements were collected on both the right and left sides.

### 2.4 Instrumentation and data collection

Haemodynamic measurements were collected using two instruments: an Innocor inert gas rebreathing device (Cosmed: The Metabolic Company, Rome, Italy) and a Finapres NOVA (Finapres Medical Systems BV, Enschede, The Netherlands). Full calibration was performed on devices daily, and ambient data calibrations were also performed prior to each subject test (mean ± SD: temperature 24.4°C ± 1.2°C, relative humidity 46.7% ± 5.8% and pressure 753.6 ± 4.2 mmHg). The inert gas rebreathing method was used to obtain non‐invasive measures of pulmonary blood flow by analysing the changing concentrations of a soluble gas (nitrous oxide, 5%) and an insoluble gas (sulfur hexafluoride, 1%) in an oxygen‐enriched air mixture over five to six breaths. The mixture was rebreathed using a bag for ∼30 s. During the rebreathe, subjects inspired and expired at a rate of 20 breaths/min, following this rhythm with a metronome (respiration at all other times was at a normal relaxed respiration rate). After each rebreathe, the gas concentration traces were inspected visually by a trained operator to ensure the correct function of the device. Further details on the inert gas rebreathing methodology can be found in the paper by Whittle et al. (2022). Finapres data (finger arterial pulse contour waveform and five‐lead ECG) were collected continuously throughout the protocol, with pressure corrected to heart level using a hydrostatic height sensor placed laterally on the mid‐coronal plane at the fifth intercostal space. At each pressure level, the Finapres pressure waveform was calibrated with a discrete blood pressure measurement using a brachial sphygmomanometer. Autonomic indices were derived from the Finapres ECG trace and beat‐to‐beat RR.

Measurements of IOP were obtained at each pressure level using a contact tonometer (IC200, iCare, Vantaa, Finland). Values presented are the mean of the central four of six measures (i.e., a trimmed mean) to account for arterial and respiratory fluctuation. OPP was calculated manually for each subject from their IOP and MAP at eye level ($\mathrm{OPP}=\mathrm{MA}P_{\mathrm{eye}}-\mathrm{IOP}$). MAP_eye_ was calculated from MAP corrected for: (1) the hydrostatic pressure difference between heart and eye level; and (2) the perpendicular distance from the mid‐coronal plane of the body to the anterior placement of the globe of the eye, using a procedure previously described by Petersen et al. (2022) and Hall and Whittle (2024).

The IJVP was obtained by manually compressing the internal jugular vein (IJV; around the C3 vertebral level) with a VeinPress (Compremium, Bern, Switzerland) manometer attached to the head of an ultrasound probe (VScan Extend, GE Healthcare, Chicago, IL, USA). The VeinPress device was zeroed prior to each measurement. Pressure was recorded at the point at which the walls of the IJV vessel were about to touch each other. When this occurred, the pressure reading was allowed to stabilize for 2 s to counter any inertial effects. Two IJVP measurements were collected at each LBNP–position–side combination, and the final IJVP in that condition was calculated as the average of the two measurements.

All other measurements of the carotid arteries and jugular veins were collected using a Butterfly iQ+ ultrasound device (Butterfly Network Inc., Burlington, MA, USA). Four separate images were obtained from each side of the subject in each experimental condition (i.e., pressure–position combination). Two images captured a transverse view of the common carotid artery (CCA) and IJV, respectively, collected ∼30 mm inferior to the CCA bifurcation point (around the C3 vertebral level) at end diastole. Two trained operators, acting independently, manually identified and circumscribed the CCA and IJV on each image to calculate *A*_CCA_ and *A*_IJV_ based on pixel count. If the two measured areas from the different operators differed by <10%, the final *A*_IJV_ in that condition was calculated as the average of the two independently measured areas. However, if the measured area differed by >10%, a third operator repeated the circumscription, and the final *A*_IJV_ in that condition was calculated as the average of the three independently measured areas.

**FIGURE 1:**
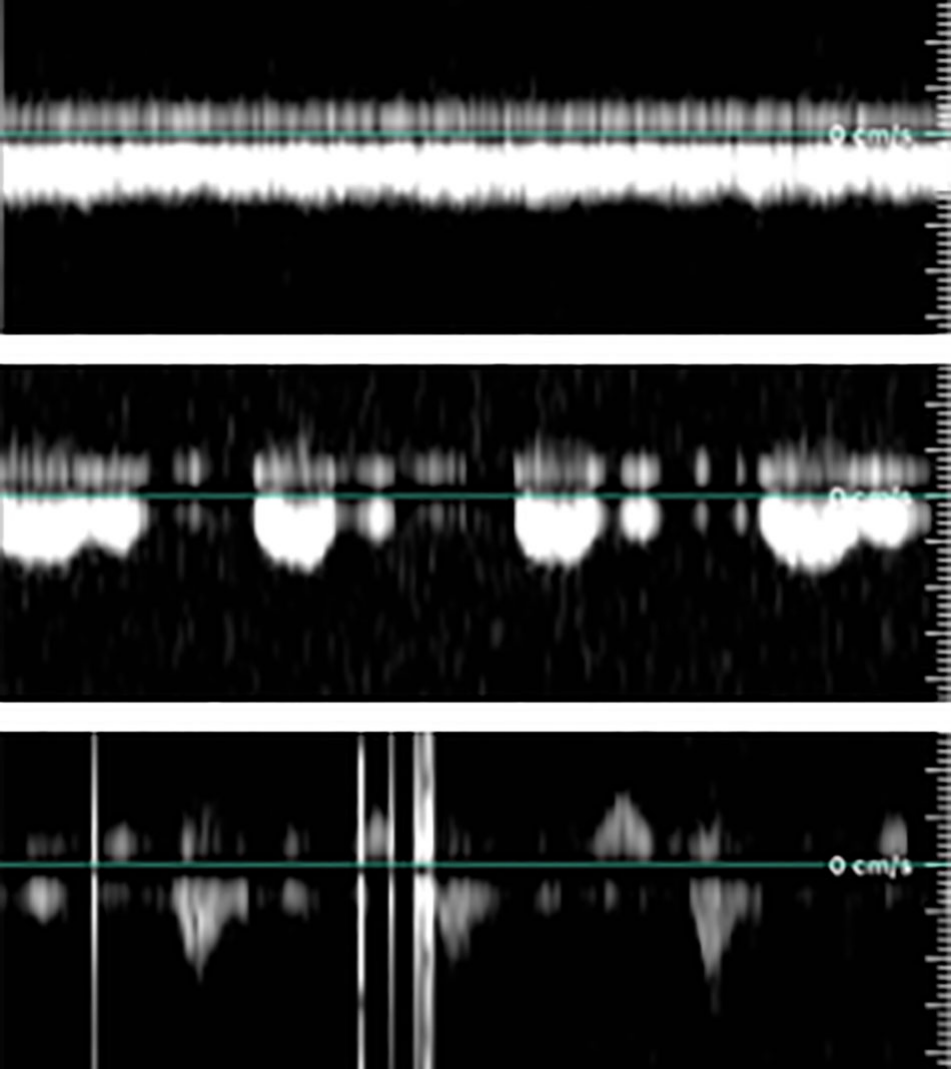
Examples of internal jugular vein blood flow velocity waveform grades found in the present study. Top, grade 1, in which forward flow that never returns to zero. Middle, grade 2, in which forward flow that can return to zero. Bottom, grade 3, with stagnant flow characterized by equal forward and retrograde flow. Flow grade regimes are as defined by Marshall‐Goebel et al. (2019).

The final two images captured spectral pulse‐wave Doppler flow of the CCA and IJV, respectively. For the CCA flow, an envelope‐tracing algorithm was applied, modified from an algorithm developed by Wadehn and Heldt (2020). The output of this algorithm was used to calculate average PSV and EDV. The IJV was binned into categories representing four flow regimes as defined by Marshall‐Goebel et al. (2019). These flow grades represent: grade 1, forward flow that never returns to zero; grade 2, forward flow that can return to zero; grade 3, stagnant flow characterized by equal forward and retrograde flow; and grade 4, predominantly retrograde flow. No instances of grade 4 flow were observed. Examples of IJV blood flow velocity waveform grades 1, 2, and 3 found in the present study are presented in Figure 1.

### 2.5 Statistical analysis

We generated dose–response curves using a Bayesian workflow in order to capture dependent structural relationships between all the variables measured via a multivariate analysis. In the next few paragraphs, we report our methodology fully and comprehensively, following the Bayesian Analysis Reporting Guidelines (BARG) given by Kruschke (2021).

All variables measured (with the exception of IJVF, described separately below) exhibited an approximately linear response to graded LBNP. A single Bayesian robust multivariate, hierarchical regression model was used to estimate the effects of LBNP, sex, and position (0° supine or 15° HDT) on all dependent variables. The multivariate model is presented in Equation 2:

$$y_{\mathrm{ip}}∼\mathrm{Student}\left(\mu_{p},\sigma_{p},\nu_{p}\right)$$

$$\;\mu_{p}=\beta_{p,0}+\beta_{p,\mathrm{LBNP}}\left(Pressure\right)+\beta_{p,\mathrm{sex}}\left(Sex\right)+\beta_{p,\mathrm{pos}}\left(Position\right)+\cdots +\gamma_{\mathrm{ip}}$$

(2) $$\gamma_{\mathrm{ip}}∼N\left(0,\sigma_{\mathrm{up}}\right)$$

where, for each dependent variable p outlined in Section 2.3, y represents the standardized response, σ represents the population‐level standard deviation, ν represents the degrees of freedom, and μ represents the mean value; μ is described further by $\beta_{0}$, $\beta_{\mathrm{LBNP}}$, $\beta_{\mathrm{sex}}$ and $\beta_{\mathrm{pos}}$, representing the coefficients for the independent variables and $\gamma_{i}$ representing a group‐level intercept for the subject i, distributed normally with mean 0 and standard deviation $\sigma_{u}$; ‘…’ represents the inclusion of any additional terms owing to interaction effects (see below). Pressure is an index variable representing the LBNP level, with 0 indicating 0 mmHg through to 5 indicating −50 mmHg, such that $\beta_{\mathrm{LBNP}}$ gives the effect size of a decrease in 10 mmHg. Sex represents a contrast‐coded variable (−0.5= female and 0.5= male), such that $\beta_{\mathrm{sex}}$ gives the effect size of the increase in $y_{p}$ in males over females. Position is a categorical variable, with zero representing 0° supine and one representing 15° HDT, such that $\beta_{\mathrm{pos}}$ gives the effect size of a change from 0° supine to 15° HDT.

Prior to constructing the dose–response curves, all dependent variables (except IJVF) were standardized to have a mean of zero and a standard deviation of 0.5. This standardization was implemented for three principal reasons. First, the prior choice was greatly simplified, because priors on the same scale could be used for all dependent variables. Second, the computational efficiency of the Bayesian calculation was greatly improved. Finally, and most importantly, this process allowed for the comparison of the magnitude of the effect sizes across different variables. Results of our modelling efforts are presented both in this standardized form and also back‐transformed to the original scales of measurement. Finally, correlations were modelled to exist between the dependent variables, p, such that the covariance matrix of all the $\sigma_{u}$ for the group‐level intercepts was described by a Cholesky decomposition.

For each dependent variable p, Bayes factors analysis of a simple univariate regression was used to determine any additional interaction effects to be included in the model, with a Bayes factor of three (i.e., substantial evidence) used as the decision rule in favour of a more complicated model (Jeffreys, 1961).

For the eight variables related to the head and neck, an additional independent contrast‐coded variable, Side (and any appropriate interactions, determined using Bayes factors), was included, such that $\beta_{\mathrm{side}}$ gives the effect size of the increase in $y_{p}$ in the right side over the left side.

In the case of IJVF and owing to the nature of the data, we implemented an ordinal logistic regression model. For this model, the dependent variable was flow grade (ranging from 1 to 3). The model used a binomial distribution with a logistic (logit) link, presented in Equation 3. Pressure, Sex, Position and Side remained as the predictor variables, and the group‐level intercept was allowed to correlate with the remainder of the dependent variables as described above. In this case, the coefficients, β, of the independent variables represent the log odds of either a grade 2 or grade 3 flow pattern with respect to a grade 1 pattern. Thus, $e^{\beta }$ represents the odds ratio (OR). The IJVF model is described further below:

$$y_{i,\mathrm{IJVF}}^{*}∼Cumulative\left(\mu_{i,\mathrm{IJVF}}\right)$$

$$\begin{array}{ccc} \mathrm{logit}\;\left(\mu_{i,\mathrm{IJVF}}\right) & = & \beta_{\mathrm{IJVF},0}+\beta_{\mathrm{IJVF},\mathrm{LBNP}}\left(Pressure\right)+\beta_{\mathrm{IJVF},\mathrm{sex}}\left(Sex\right)\\ & & +\;\beta_{\mathrm{IJVF},\mathrm{pos}}\left(Position\right)+\beta_{\mathrm{IJVF},\mathrm{side}}\left(Side\right)+\gamma_{i,\mathrm{IJVF}} \end{array}$$

(3) $$\gamma_{i,\mathrm{IJVF}}∼N\left(0,\sigma_{u,\mathrm{IJVF}}\right)$$

where $y_{i,\mathrm{IJVF}}^{*}$ is the latent variable for the IJV blood flow velocity waveform pattern for the subject i; $\mu_{i,\mathrm{IJVF}}$ is the linear predictor (with a logit link function); $\beta_{\mathrm{IJVF},0}$, $\beta_{\mathrm{IJVF},\mathrm{LBNP}}$, $\beta_{\mathrm{IJVF},\mathrm{sex}}$, $\beta_{\mathrm{IJVF},\mathrm{pos}}$ and $\beta_{\mathrm{IJVF},\mathrm{side}}$ are the coefficients for the intercept, LBNP Pressure, Sex (male or female), Position (0° supine or 15° HDT) and Side (right or left), respectively; $\gamma_{i,\mathrm{IJVF}}$ is the group‐level intercept for the subject i; and $\sigma_{u,\mathrm{IJVF}}$ is the standard deviation of the group‐level intercept. Owing to significant heterogeneity (i.e., the variance of the data increasing with stronger LBNP), a distributional regression model was used such that $\sigma_{\mathrm{IJVP}}$ was allowed to vary with the pressure level as indicated in Equation 4:

(4) $$\log\left(\sigma_{\mathrm{IJVP}}\right)=\zeta_{0,\mathrm{IJVP}}+\zeta_{\mathrm{LBNP},\mathrm{IJVP}}\left(Pressure\right)$$

where $\zeta_{0,\mathrm{IJVP}}$ and $\zeta_{\mathrm{LBNP},\mathrm{IJVP}}$ are the intercept and slope of the log of the $\sigma_{\mathrm{IJVP}}$, respectively.

Finally, in order to determine the multivariate relationship between the variables measured and the subject characteristics, standardized Age, Height, Weight and BMI were added to the multivariate regression model as dependent variables, defined as a unique group‐varying intercept in the form of Equation 5:

$$\;y_{i,\mathrm{chars}}=\gamma_{i,\mathrm{chars}}\;$$

(5) $$\gamma_{i,\mathrm{chars}}∼N\left(0,\sigma_{u,\mathrm{chars}}\right)$$

where $y_{i,\mathrm{chars}}$ is the characteristic (Age, Height, Weight or BMI) of subject i; and $\gamma_{i,\mathrm{chars}}$ is a group‐level intercept with a standard deviation $\sigma_{u,\mathrm{chars}}$.

Table 2 presents the form of the regression model for all dependent variables, p.

**TABLE 2:** Bayesian multivariate regression model used to construct the dose–response curves for the cardiovascular response to LBNP.

Measure	Distribution	Main effects	Additional effects^a^
HR	Student	Pressure, Sex, Position	–
SV	Student	Pressure, Sex, Position	Pressure×Sex
CO	Student	Pressure, Sex, Position	–
${\dot{V}}_{O_{2}}$	Student	Pressure, Sex, Position	Sex×Position
SBP	Student	Pressure, Sex, Position	–
DBP	Student	Pressure, Sex, Position	–
RPP^b^	N	Pressure, Sex, Position	–
MO	Student	Pressure, Sex, Position	–
TPR	Student	Pressure, Sex, Position	–
SI	Student	Pressure, Sex, Position	–
CI	Student	Pressure, Sex, Position	–
SDNN	Student	Pressure, Sex, Position	–
HRVTi	Student	Pressure, Sex, Position	–
RMSDD	Student	Pressure, Sex, Position	–
BRS	Student	Pressure, Sex, Position	–
IOP	Student	Pressure, Sex, Position, Side	–
OPP	Student	Pressure, Sex, Position, Side	–
*A*_IJV_	Student	Pressure, Sex, Position, Side	–
IJVP	Student	Pressure, Sex, Position, Side	log(σ)∼Pressure
IJVF^c^	Cumulative	Pressure, Sex, Position, Side	–
*A*_CCA_	Student	Pressure, Sex, Position, Side	–
PSV	Student	Pressure, Sex, Position, Side	–
EDV	Student	Pressure, Sex, Position, Side	Pressure×Sex, Sex×Position, Sex×Side
Age^d^	–	–	–
Height^d^	–	–	–
Weight^d^	–	–	–
BMI^d^	–	–	–

*Note*: Distributions, main effects and additional effects (interaction or distributional parameters) for each dependent variable are included in the table. All dependent variables are combined into a single matrix and analysed using a single, large regression model, as detailed in the main text.

Abbreviations: *A*_CCA_, common carotid artery area; *A*_IJV_, internal jugular vein cross‐sectional area; BRS, baroreflex sensitivity; CI, cardiac index; CO, cardiac output; DBP, diastolic blood pressure; EDV, end‐diastolic velocity; HR, heart rate; HRVTi, heart rate variability triangular index; IJVP, internal jugular vein pressure; IOP, intraocular pressure; MO, myocardial oxygen supply:demand index; OPP, ocular perfusion pressure; PSV, peak systolic velocity; RMSDD, root mean square of direct differences of NN intervals; RPP, rate–pressure product; SBP, systolic blood pressure; SDNN, standard deviation of NN intervals; SI, stroke index; SV, systolic velocity; TPR, total peripheral resistance; ${\dot{V}}_{O_{2}}$, oxygen consumption.

^a^ Interactions or distributional parameters.

^b^ Bayes factor analysis strongly favoured a Gaussian distribution over a robust Student distribution for RPP.

^c^ As described in the main text, IJVF flow pattern was modelled as an ordinal logistic regression with a logit link.

^d^ Subject characteristics modelled as a unique group‐level intercept.

Weakly informative priors were chosen across all parameters, and they are summarized in Table 3. Normal priors were used for all β and ζ. Following the recommendations of Gelman (2006), half‐Cauchy distributions were used for all σ and $\sigma_{u}$. Gamma priors were used for all ν. The covariance matrix, Σ, was assigned a Lewandowski–Kurowicka–Joe (LKJ) prior (Lewandowski et al., 2009; van Zundert et al., 2022). Prior predictive checks were conducted to ensure that the priors generated credible estimates.

**TABLE 3:** Weakly informative priors used for the Bayesian multivariate dose–response model.

Group	Prior	Comment
$\beta_{0}$	N(0,1)	Intercept
β	N(0,1)	Slope^a^
$\zeta_{0}$	N(0,1)	Distributional intercept parameter
$\zeta_{\mathrm{LBNP}}$	N(0,1)	Distributional slope parameter
ν	Γ(2,0.1)	Degrees of freedom
σ	$Cauchy^{+}\left(0,1\right)$	Population variance
$\sigma_{u}$	$Cauchy^{+}\left(0,1\right)$	Group variance
L, R	LKJcorr(1)	Correlation structure^b^

*Note*: The priors apply to all relevant standardized dependent variables, p, in line with the model formulae outlined in Table 2.

^a^ Applies to $\beta_{\mathrm{LBNP}}$, $\beta_{\mathrm{sex}}$, $\beta_{\mathrm{pos}}$, $\beta_{\mathrm{side}}$ (where relevant), and any interactions.

^b^ Lewandowski–Kurowicka–Joe (LKJ) Cholesky correlation distribution with shape parameter η=1.

The model was fitted via Markov chain Monte Carlo using Stan version 2.26.1 (Stan Development Team, 2022), R version 4.4.2 (R Core Team, 2025), and the brms package (Bürkner, 2017; Bürkner, 2018; Bürkner, 2021). Stan is a probabilistic programming platform for statistical modelling and high‐performance statistical computation, where Hamilton Monte Carlo sampling is performed using a no‐U‐turn sampler toexplore posteriors in models efficiently. The model was sampled using 20 000 draws (1000 burn‐in) in each of four chains. In the fitted model, chain diagnostics were inspected visually to ensure good mixing, with all $\hat{R}$ values <1.01 and all effective chain lengths >5000 (Gelman & Rubin, 1992). Posterior predictive checks were conducted to ensure that the posterior estimates approximated the data distribution. Pareto‐smoothed leave‐one‐out cross‐validation was conducted to ensure accurate model predictive power (Vehtari et al., 2024). All posterior summaries are given using the maximum a posteriori estimate and the 89% highest density interval [also known as the 89% credible interval (89% CrI)].

We also report evidence of existence and significance of effects, where existence is defined as ‘the consistency of an effect in one particular direction (i.e., positive or negative), without any assumptions or conclusions as to its size, importance, relevance or meaning’ (Makowski et al., 2019) and significance is ‘being worthy of attention or important’ (Makowski et al., 2019). Evidence for the existence of an effect is presented using the probability of direction (pd), defined as the proportion of the posterior distribution that is of the same sign as its median (from 50% to 100%) (Makowski et al., 2019). Evidence for the significance of an effect (as an analogy to a frequentist p‐value) is presented as the percentage of the full posterior for any particular effect (Pressure, Sex, Position, or Side) inside a region of practical equivalence (ROPE), given as ${\%}_{\mathrm{ROPE}}$. For the majority of parameters, the ROPE is defined as [−0.05,0.05] on a normalized scale (or $\left[-0.1sd\left(y_{p}\right),0.1sd\left(y_{p}\right)\right]$ on the original scale of measurement) (Cohen, 1988; Kruschke & Liddell, 2018). For the log odds parameters related to IJVF, the ROPE is defined as $\left[-0.1\pi /\sqrt{3},0.1\pi /\sqrt{3}\right]$ (Kruschke & Liddell, 2018). In general, if >95% of the full posterior distribution is inside the ROPE, this can be interpreted as strong evidence in favour of the null hypothesis (no effect), whereas if <5% of the full posterior distribution is inside the ROPE, this can be interpreted as strong evidence of an effect (Schwaferts & Augustin, 2020). Both pd and ${\%}_{ROPE}$ must be interpreted together. For example, a posterior distribution that is centralized about zero but very wide might have a low ${\%}_{ROPE}$, whilst pd≈50% (half the distribution is positive and half of the distribution is negative). This implies that, although there might be an effect, there is little evidence regarding whether that effect is positive or negative.

## 3 RESULTS

### 3.1 Raw data

#### 3.1.1 Systemic haemodynamic response

**FIGURE 2:**
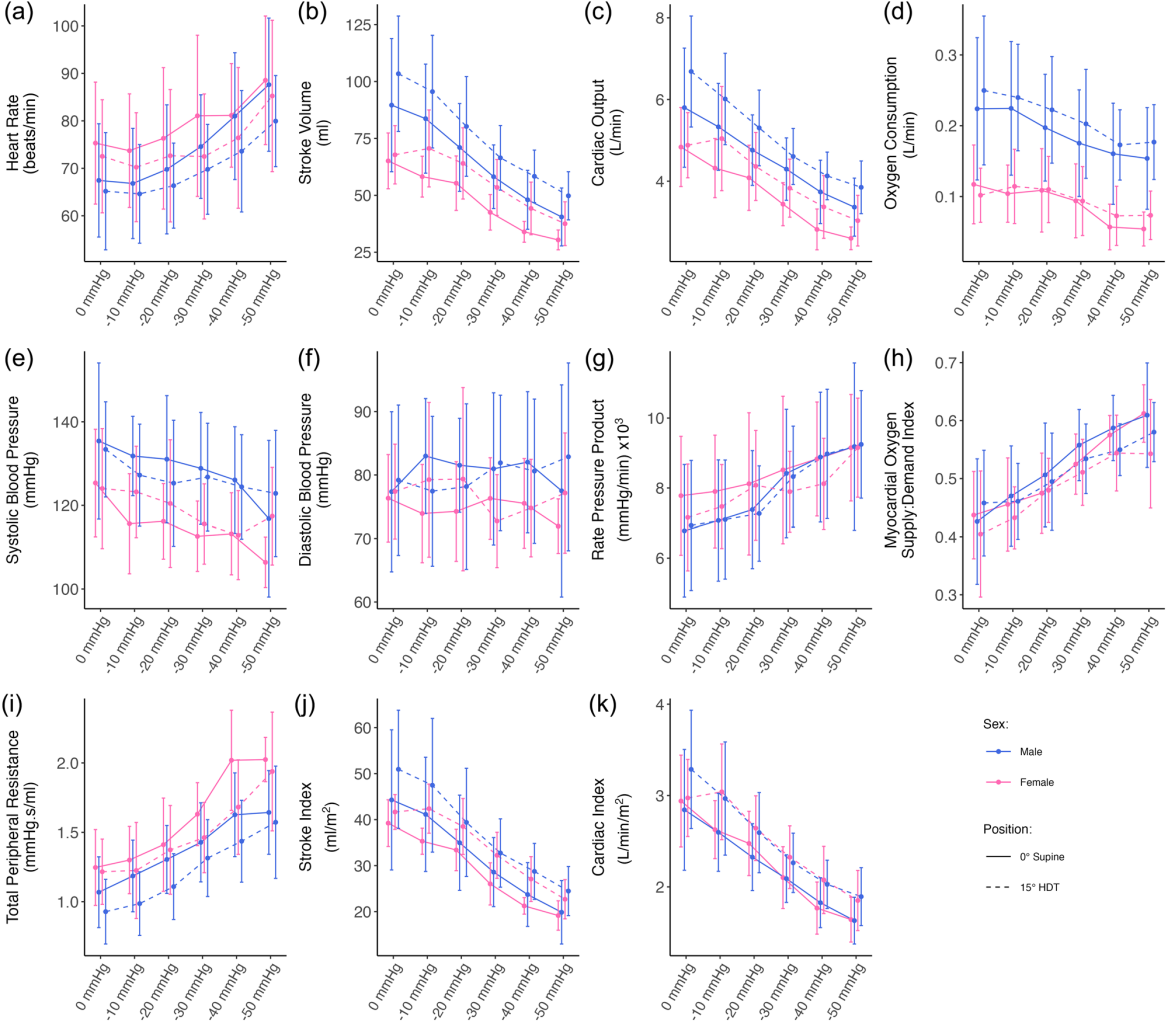
Systemic haemodynamic variables as a function of LBNP in 0° supine (continuous line) and 15° HDT (dashed line) positions, collected on 24 subjects (12 male and 12 female). Measurements were collected at 0, −10, −20, −30, −40 and −50 mmHg of LBNP. Data are presented as means ± SD at each pressure level. (a) HR; (b) SV; (c) CO; (d) ${\dot{V}}_{O_{2}}$; (e) SBP; (f) DBP; (g) RPP; (h) MO; (i) TPR; (j) SI; (k) CI. Abbreviations: CI, cardiac index; CO, cardiac output; DBP, diastolic blood pressure; HDT, head‐down tilt; HR, heart rate; LBNP, lower‐body negative pressure; MO, myocardial oxygen supply:demand index; RPP, rate–pressure product; SBP, systolic blood pressure; SI, stroke index; SV, stroke volume; TPR, total peripheral resistance; ${\dot{V}}_{O_{2}}$, oxygen consumption.

Figure 2 shows the evolution of systemic haemodynamic parameters (mean ± SD) as a function of LBNP (the seated baseline has been removed for clarity). All measured variables follow an approximately linear trend with respect to LBNP.

In males, HR (Figure 2a) increases from 67.5 ± 3.4 beats/min at 0 mmHg of LBNP to 87.6 ± 4.1 beats/min at −50 mmHg of LBNP in 0° supine and from 65.2 ± 3.6 beats/min at 0 mmHg of LBNP to 79.9 ± 2.8 beats/min at −50 mmHg of LBNP in 15° HDT. In general, female subjects have an HR that is 2.6 beats/min (89% CrI: −4.4 to 9.2 beats/min) higher than males (pd=72.16%, ${\%}_{ROPE}=21.91\%$). The SV (Figure 2b) and CO (Figure 2c) decrease at an average rate (males and females together) of 8.8 mL (89% CrI: 8.3 to 9.4 mL) and 0.48 L/min (89% CrI: 0.45 to 0.50 L/min), respectively, for every 10 mmHg increase in LBNP strength (more negative). In absolute values, SV and CO are higher in males, with respect to the females, by 23.3 mL (89% CrI: 15.5 to 31.6 mL) and 0.57 L/min (89% CrI: 0.16 to 1.00 L/min), respectively. However, when variables are indexed by body surface area, cardiac index (CI; Figure 2k) is equivalent in males and females, decreasing by 0.26 L/min/m^2^ (89% CrI: 0.24 to 0.27 L/min/m^2^, males and females together) per 10 mmHg increase in LBNP. Stroke index (SI; Figure 2j) is still marginally higher in male subjects by, on average, 3.5 mL/m^2^ (89% CrI: 0.0 to 7.5 mL/m^2^).

The SBP (Figure 2e), which is higher in males by 7.4 mmHg (89% CrI: 1.1 to 13.7 mmHg), decreases slightly at an average rate of 2.1 mmHg (89% CrI: 1.5 to 2.6 mmHg) per 10 mmHg increase in LBNP. However, DBP (Figure 2f) appears to hold a relatively constant value, with no clear trend.

The RPP (Figure 2g), myocardial oxygen supply:demand index (MO; Figure 2h) and TPR (Figure 2i) increase linearly across the range of LBNP values considered. There is no difference in MO between male and female subjects (0.01, 89% CrI: −0.03 to 0.06); however, RPP and TPR appear to be slightly higher in females (for RPP, 290 mmHg/min higher, 89% CrI −640 to 1150 mmHg/min, pd=67.61%, ${\%}_{ROPE}=23.35\%$; for TPR, 0.15 mmHg s/mL higher, 89% CrI −0.01 to 0.30 mmHg s/mL, pd=93.55%, ${\%}_{ROPE}=10.08\%$). In RPP, this is most noticeable at lower pressure levels and is likely to be driven by the higher resting HR in female subjects.

The largest difference between males and females appears in ${\dot{V}}_{O_{2}}$, where males consume twice as much oxygen as females. Males have an average ${\dot{V}}_{O_{2}}$ of 0.22 ± 0.03 L/min at 0 mmHg of LBNP, and this value decreases to 0.15 ± 0.02 L/min at −50 mmHg of LBNP (in 0° supine). Females have an average ${\dot{V}}_{O_{2}}$ of 0.12 ± 0.02 L/min at 0 mmHg of LBNP, and this value decreases to 0.05 ± 0.01 L/min at −50 mmHg of LBNP (0° supine). Overall, the effect of LBNP, sex, or position (0° supine or 15° HDT) appears minimal. This result is supported by evidence from our previous tilt studies (Whittle et al., 2022), which noted no effect of tilt angle on ${\dot{V}}_{O_{2}}$.

#### 3.1.2 Autonomic response

**FIGURE 3:**
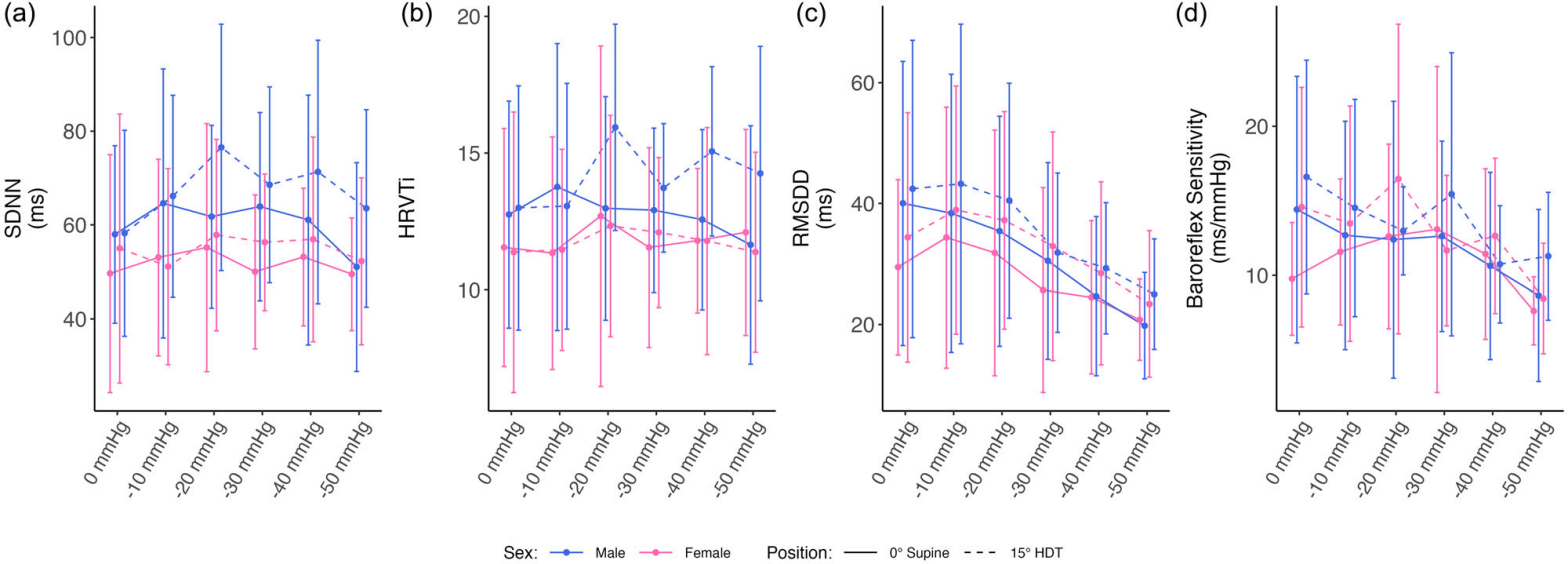
Autonomic variables as a function of LBNP in 0° supine (continuous line) and 15° HDT (dashed line) positions, collected on 24 subjects (12 male and 12 female). Measurements were collected at 0, −10, −20, −30, −40 and −50 mmHg of LBNP. Data are presented as means ± SD at each pressure level. (a) SDNN; (b) HRVTi; (c) RMSDD; (d) BRS. Abbreviations: BRS, baroreflex sensitivity; HDT, head‐down tilt; HRVTi, heart rate variability triangular index; LBNP, lower‐body negative pressure; RMSDD, root mean square of direct differences of NN intervals; SDNN, standard deviation of NN intervals.

Figure 3 shows the evolution of autonomic parameters (mean ± SD) as a function of LBNP (the seated baseline has been removed for clarity).

Broadly, there is minimal effect of LBNP on overall HR variability, as evidenced by a minimal significant effect of LBNP on SDNN (Figure 3a) or HRVTi (Figure 3b). In contrast, the overall balance of sympathetic and vagal activity is clearly altered by LBNP. In particular, RMSDD (Figure 3c), a marker of vagal activity, decreases from 35.5 ± 3.0 ms at 0 mmHg of LBNP to 22.4 ± 1.5 ms at −50 mmHg of LBNP (average of both sexes and both positions), a reduction of 2.8 ms (89% CrI: 2.2 to 3.3 ms) per 10 mmHg increase in LBNP. Finally, BRS (Figure 3d) decreases slightly with LBNP, from 13.8 ± 1.1 ms/mmHg at 0 mmHg to 9.2 ± 0.7 ms/mmHg at −50 mmHg (average of both sexes and both positions). This is an average decrease of 0.8 ms/mmHg (89% CrI: 0.6 to 1.1 ms/mmHg) per 10 mmHg increase in LBNP. This reduction in sensitivity is likely to be related to the reduction in blood flow and pressure in the carotid sinus and aortic arch with increased LBNP, as blood is pooled in the lower body.

#### 3.1.3 Head/neck response

**FIGURE 4:**
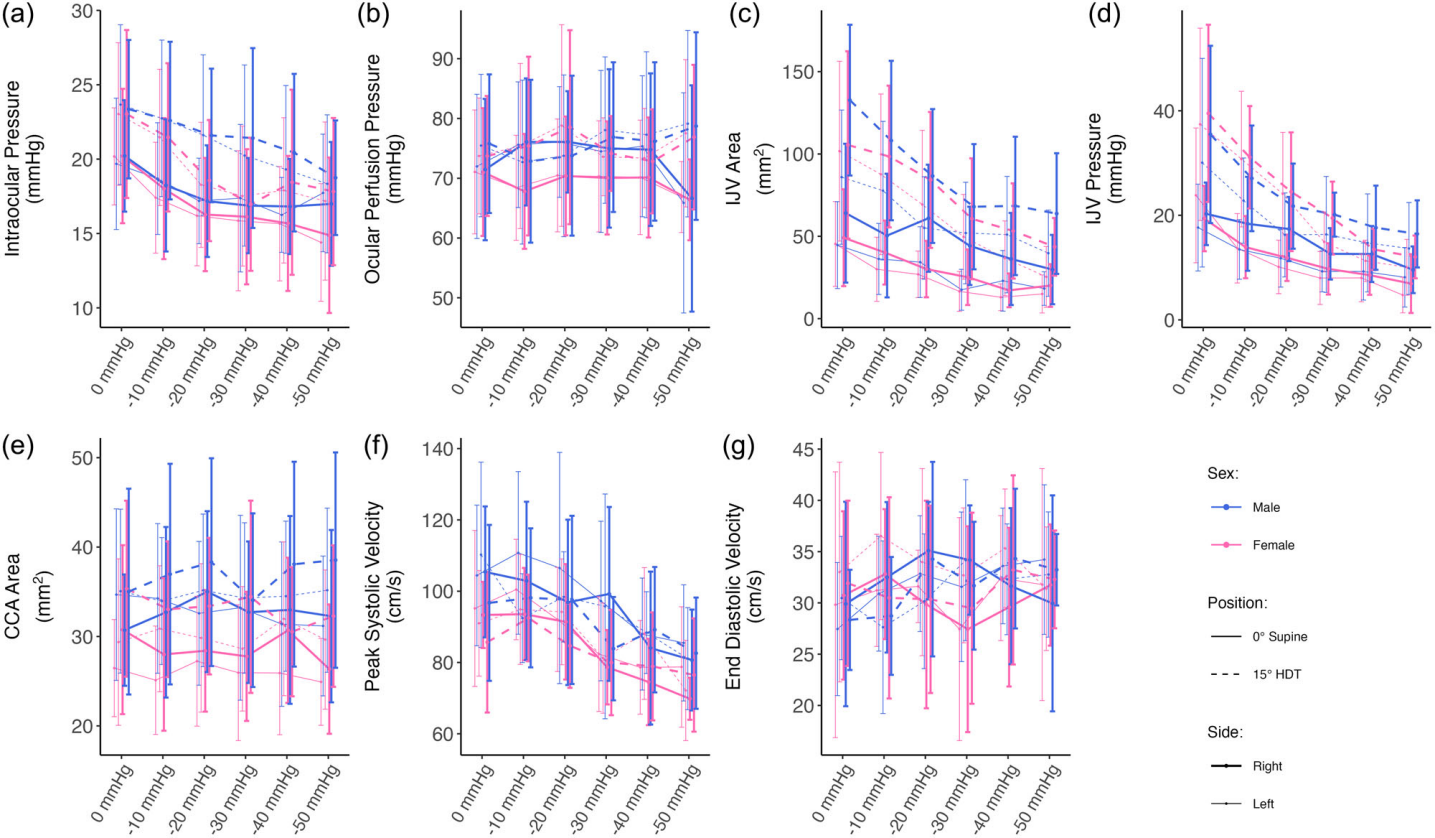
Head/neck variables as a function of LBNP in 0° supine (continuous line) and 15° HDT (dashed line) positions, collected on 24 subjects (12 male and 12 female). Thick lines represent the right side and thin lines the left side. Measurements were collected at 0, −10, −20, −30, −40 and −50 mmHg of LBNP. Data are presented as means ± SD at each pressure level. (a) IOP; (b) OPP; (c) *A*_IJV_; (d) IJVP; (e) *A*_CCA_; (f) PSV; (g) EDV. Abbreviations: *A*_CCA_, common carotid artery area; *A*_IJV_, internal jugular vein cross‐sectional area; EDV, end‐diastolic velocity; HDT, head‐down tilt; IJVP, internal jugular vein pressure; IOP, intraocular pressure; LBNP, lower‐body negative pressure; OPP, ocular perfusion pressure; PSV, peak systolic velocity.

**FIGURE 5:**
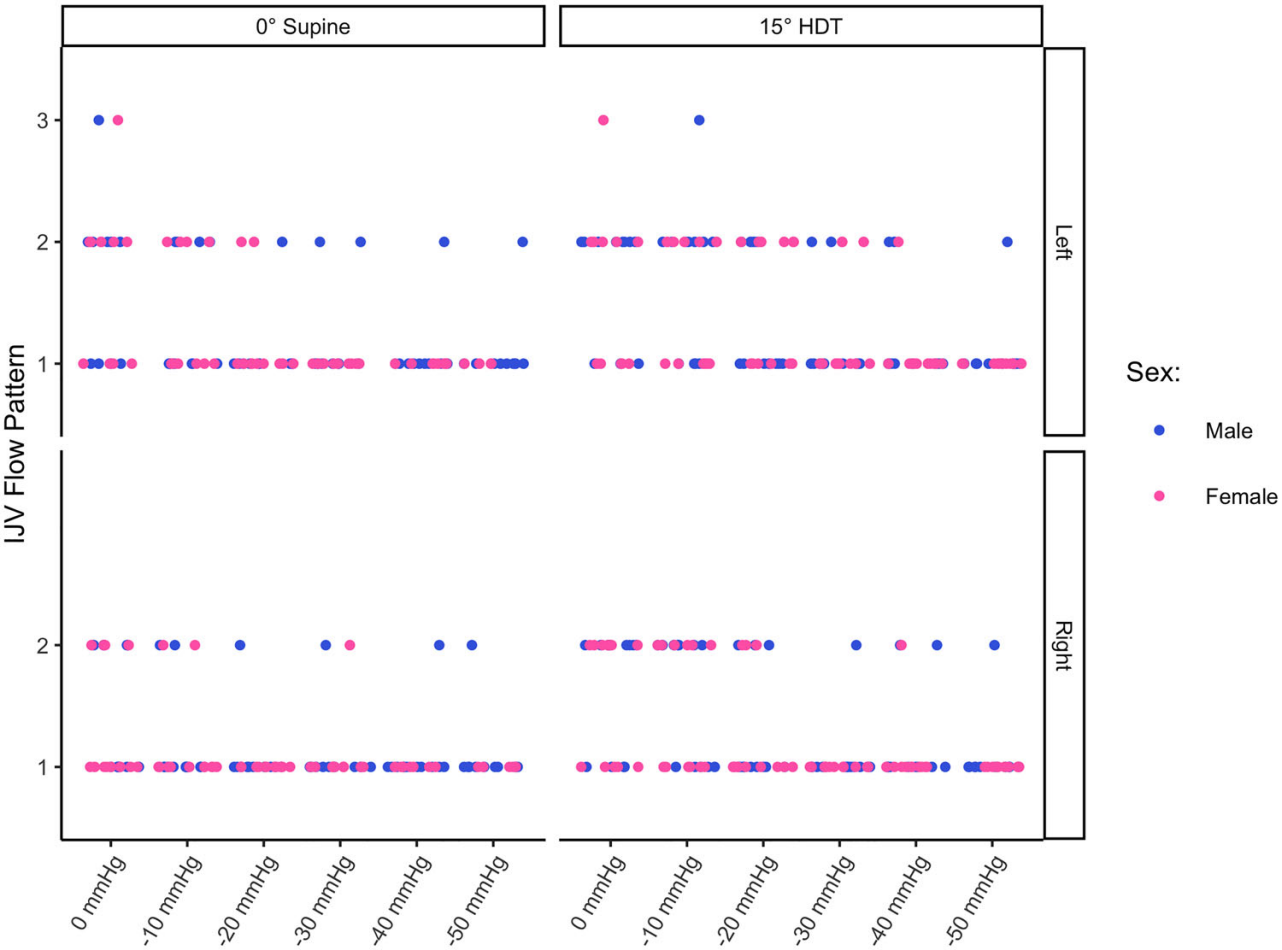
IJV blood flow velocity waveform pattern as a function of LBNP in 0° supine (left column) and 15° HDT positions (right column), on the left (top row) and right (bottom row) sides. Data were collected on 24 subjects (12 male and 12 female). Flow grade patterns are described in the main text, illustrated in Figure 1, and taken from Marshall‐Goebel et al. (2019). Abbreviations: HDT, head‐down tilt; IJV, internal jugular vein; LBNP, lower‐body negative pressure.

Figure 4 shows the evolution of head/neck parameters (mean ± SD), excluding IJVF, as a function of LBNP. Figure 5 shows the IJV blood flow velocity waveform pattern as a function of LBNP. The seated baseline data have been removed for clarity.

There appears to be little difference between the right and left sides (thick and thin lines, respectively) across all the variables considered. In the 15° HDT position, IOP (Figure 4a) is 2.8 mmHg (89% CrI: 2.3 to 3.2 mmHg) higher than IOP in the 0° supine position. The IOP also decreases linearly from 20.1 ± 0.6 mmHg at 0 mmHg of LBNP to 16.4 ± 0.7 mmHg at −50 mmHg of LBNP (0° supine, average of both sexes and both sides). In 15° HDT, IOP decreases from 23.3 ± 0.7 mmHg at 0 mmHg of LBNP to 18.1 ± 0.7 mmHg at −50 mmHg of LBNP (both sexes and sides averaged). In contrast, there appears to be no significant effect of LBNP on OPP (Figure 4b), with the response remaining relatively constant across all LBNP levels considered [there is even a slight increase in 15° HDT from 74.7 ± 1.5 mmHg at 0 mmHg of LBNP to 78.1 ± 2.1 mmHg at −50 mmHg of LBNP (male and female, both sides)].

The common carotid artery area (CCA, Figure 4e) does not show a significant effect with LBNP, although we find a small effect of position. The average *A*_CCA_ at 0° supine is 30.3 ± 0.5 mm (Hargens & Richardson, 2009), which increases to 33.7 ± 0.6 mm (Hargens & Richardson, 2009) in 15° HDT (average of all pressure levels, sexes and sides). There is minimal change in EDV of the CCA with LBNP (Figure 4g); however, the PSV (Figure 4f) decreases from 97.7 ± 2.1 cm/s at 0 mmHg of LBNP to 79.1 ± 1.7 cm/s at −50 mmHg of LBNP (average of both sides, both sexes and both positions), indicating an overall decrease in the pulse velocity with LBNP strength (more negative).

Finally, the IJV exhibits a strong response to LBNP, with decreases of 8.4 mm (89% CrI: 7.3 to 9.7 mm; Hargens & Richardson, 2009) per 10 mmHg of LBNP in area (*A*_IJV_; Figure 4c) and 2.6 mmHg (89% CrI: 2.3 to 2.9 mmHg) per 10 mmHg of LBNP in pressure (IJVP; Figure 4d). Both *A*_IJV_ and IJVP increase in the 15° HDT position with respect to the 0° supine position: *A*_IJV_ increases by 39.5 mm (89% CrI: 35.8 to 43.4 mm; Hargens & Richardson, 2009) and IJVP by 7.2 mmHg (89% CrI: 6.3 to 8.2 mmHg). However, the results do not show an effect of side on *A*_IJV_. These results contrast with our previous data from tilt studies (Whittle & Diaz‐Artiles, 2023). However, this is not necessarily surprising, because the larger differences in *A*_IJV_ between the left and right sides only begin to appear in HDT and continue to be amplified at larger tilt angles (30° and 45° HDT). In the present study, subjects remained at 15° HDT during the entire intervention, which can explain the lack of significant differences in *A*_IJV_ between the right and left sides. With respect to the IJV blood flow velocity waveform patterns, Figure 5 reveals that, although the majority of observed flows are at grade 1, we find more instances of grade 2 flow at lower LBNP levels (and even two cases of grade 3 flow stagnation, both appearing in the left IJV). Overall, our results suggest that LBNP is effective at reducing the instances of grade 2 flow.

### 3.2 Dose response

From the fitted dose–response model, we can extract and visualize the posterior draws for the effect size of each individual main effect (Pressure, Sex, Position, or Side) on each dependent variable p. Given that all dependent variables in the model were standardized, the effect sizes can be compared across different variables. Sections 4, 4.1, 3.2.3, and 3.2.4 consider the effect sizes of Pressure, Sex, Position, and Side, respectively, from the fitted dose–response curves.

Table 4 presents the fitted parameters from the dose–response curves, back‐transformed from the standardized posterior distributions into their original units. Finally, Table 5 presents the pd and ${\%}_{ROPE}$ for each of the four main effects (Pressure, $\beta_{\mathrm{LBNP}}$; Sex, $\beta_{\mathrm{sex}}$; Position, $\beta_{\mathrm{pos}}$; and Side, $\beta_{\mathrm{side}}$) for all dependent variables. The pd and ${\%}_{ROPE}$ are invariant of the scale used (normalized or original), because the ROPE scales with the dependent variable.

**TABLE 4:** Posterior estimates for dose–response curves fitted to all measured parameters.

Parameter	$\beta_{0}$	$\beta_{\mathrm{LBNP}}$ ^a^	$\beta_{\mathrm{sex}}$ ^b^	$\beta_{\mathrm{pos}}$ ^c^	$\beta_{\mathrm{side}}$ ^d^	Additional^e^	σ	$\sigma_{u}$	ν
HR (beats/min)	70.0 (67.0, 73.0)	2.9 (2.5, 3.2)	−2.6 (−9.2, 4.4)	−4.9 (−6.0, −3.7)	–	–	5.3 (4.9, 5.9)	9.0 (7.4, 11.1)	14.6 (5.9, 42.9)
SV (mL)	78.6 (75.1, 82.1)	−8.8 (−9.4, −8.3)	23.3 (15.5, 31.6)	8.9 (7.3, 10.5)	–	−3.9 (−5.0, −3.0)^f^	7.0 (6.0, 8.1)	9.4 (7.8, 11.9)	4.6 (2.7, 11.5)
CO (L/min)	5.33 (5.14, 5.51)	−0.48 (−0.50, −0.45)	0.57 (0.16, 1.00)	0.39 (0.30, 0.48)	–	–	0.37 (0.32, 0.42)	0.54 (0.45, 0.68)	4.1 (2.6, 6.6)
${\dot{V}}_{O_{2}}$ (L/min)	0.17 (0.16, 0.19)	−0.01 (−0.01, −0.01)	0.06 (0.03, 0.10)	0.01 (0.01, 0.02)	–	0.02 (0.01, 0.03)^g^	0.02 (0.02, 0.03)	0.05 (0.04, 0.06)	3.2 (2.2, 5.0)
SBP (mmHg)	128.1 (124.9, 131.0)	−2.1 (−2.6, −1.5)	7.4 (1.1, 13.7)	−0.3 (−2.3, 1.5)	–	–	8.6 (7.3, 9.6)	7.6 (6.1, 10.1)	6.8 (3.0, 21.9)
DBP (mmHg)	77.9 (75.3, 80.4)	0.3 (−0.2, 0.8)	3.7 (−1.5, 9.0)	−0.5 (−1.9, 1.1)	–	–	6.8 (5.9, 7.6)	6.4 (5.1, 8.4)	6.8 (3.4, 18.6)
RPP (mmHg/min)	7260 (6850, 7670)	410 (360, 460)	−290 (−1150, 640)	−310 (−480, −130)	–	–	860 (800, 930)	1130 (940, 1440)	–
MO (−)	0.44 (0.42, 0.46)	0.03 (0.03, 0.03)	0.01 (−0.03, 0.06	−0.02 (−0.02, −0.01)	–	–	0.04 (0.03, 0.04)	0.05 (0.04, 0.07)	4.0 (2.6, 7.0)
TPR (mmHg s/mL)	1.12 (1.05, 1.19)	0.14 (0.13, 0.15)	−0.15 (−0.30, 0.01)	−0.12 (−0.16, −0.09)	–	–	0.16 (0.14, 0.18)	0.20 (0.16, 0.25)	8.4 (3.6, 30.2)
SI (mL/m^2^)	42.2 (40.4, 44.0)	−4.7 (−5.0, −4.4)	3.5 (−0.0, 7.5)	5.0 (4.1, 5.9)	–	–	4.0 (3.4, 4.5)	4.7 (3.8, 6.0)	5.5 (2.7, 16.2)
CI (L/min/m^2^)	2.91 (2.81, 3.00)	−0.26 (−0.27, −0.24)	0.07 (−0.13, 0.28)	0.20 (0.16, 0.25)	–	–	0.20 (0.18, 0.23)	0.27 (0.22, 0.34)	4.7 (2.9, 9.4)
SDNN (ms)	54.6 (49.7, 60.1)	−0.2 (−0.9, 0.6)	12.2 (0.6, 23.2)	6.5 (4.1, 8.7)	–	–	9.8 (8.6, 11.1)	14.4 (11.5, 18.1)	4.6 (2.8, 8.6)
HRVTi (−)	11.9 (10.9, 13.0)	0.0 (−0.1, 0.2)	1.6 (−0.6, 3.7)	0.8 (0.3, 1.3)	–	–	2.4 (2.1, 2.6)	2.7 (2.1, 3.5)	10.1 (4.1, 32.6)
RMSDD (ms)	34.8 (30.8, 39.1)	−2.8 (−3.3, −2.2)	3.6 (−5.1, 13.0)	5.0 (3.3, 6.8)	–	–	6.9 (5.9, 8.1)	11.4 (9.3, 14.7)	3.7 (2.2, 6.6)
BRS (ms/mmHg)	12.9 (11.2, 14.7)	−0.8 (−1.1, −0.6)	0.6 (−3.1, 4.5)	2.3 (1.5, 3.1)	–	–	3.3 (2.9, 3.8)	4.6 (3.5, 6.2)	3.8 (2.5, 6.2)
IOP (mmHg)	19.8 (12.4, 26.9)	−1.0 (−1.1, −0.8)	0.4 (−3.3, 4.1)	2.8 (2.3, 3.2)	0.7 (−14.3, 14.2)	–	1.9 (1.6, 2.2)	4.7 (3.7, 6.2)	5.2 (2.6, 14.0)
OPP (mmHg)	72.7 (55.0, 89.7)	0.5 (−0.0, 0.9)	2.1 (−4.6, 8.4)	2.4 (1.0, 4.1)	−1.5 (−35.7, 33.2)	–	6.6 (5.7, 7.6)	8.2 (6.6, 10.5)	4.7 (2.6, 11.8)
*A*_IJV_ (mm^2^)	52.5 (−2.9, 117.6)	−8.4 (−9.7, −7.3)	18.1 (−3.1, 38.7)	39.5 (35.8, 43.4)	7.1 (−116.9, 122.1)	–	14.1 (11.9, 16.7)	27.1 (21.3, 35.5)	2.8 (1.9, 4.6)
IJVP (mmHg)	19.2 (2.9, 36.4)	−2.6 (−2.9, −2.3)	2.7 (−0.9, 6.5)	7.2 (6.3, 8.2)	1.9 (−32.6, 33.9)	−0.20 (−0.27, −0.13)^h^	$\;\sigma_{0}=$ 3.7 (3.2, 4.3)	4.5 (3.4, 6.0)	3.0 (2.0, 4.7)
IJVF (−)^i^	1.08 (−0.44, 2.52)	−1.21 (−1.62, −0.92)	0.17 (−1.11, 1.58)	1.81 (1.08, 2.57)	−0.02 (−1.62, 1.58)	–	–	2.97 (1.94, 4.69)	–
	12.83 (8.10, 21.77)								
*A*_CCA_ (mm^2^)	31.0 (18.2, 44.6)	−0.0 (−0.3, 0.3)	1.6 (−5.2, 8.7)	3.6 (2.7, 4.6)	−1.2 (−27.0, 24.9)	–	4.1 (3.5, 4.7)	8.9 (6.9, 11.8)	4.9 (2.7, 12.7)
PSV (cm/s)	98.0 (68.6, 125.2)	−3.8 (−4.6, −3.1)	9.6 (0.2, 19.1)	−1.7 (−4.2, 1.0)	4.3 (−52.7, 59.4)	–	11.8 (10.6, 13.0)	11.7 (9.0, 15.6)	11.7 (4.7, 37.0)
EDV (cm/s)	30.5 (19.9, 42.2)	0.2 (−0.1, 0.6)	1.0 (−10.6, 12.3)	0.1 (−1.0, 1.4)	0.3 (−22.1, 22.1)	0.8 (0.1, 1.5)^j^	4.8 (4.1, 5.5)	3.8 (2.8, 5.8)	3.4 (2.3, 5.6)
						−3.4 (−5.7, −0.8)^k^			
						0.5 (−22.0, 22.0)^j^			

*Note*: Data are presented as the maximum a posteriori (89% credible interval).

Abbreviations: *A*_CCA_, common carotid artery area; *A*_IJV_, internal jugular vein cross‐sectional area; BRS, baroreflex sensitivity; CI, cardiac index; CO, cardiac output; DBP, diastolic blood pressure; EDV, end‐diastolic velocity; HR, heart rate; HRVTi, heart rate variability triangular index; IJVP, internal jugular vein pressure; IOP, intraocular pressure; LBNP, lower‐body negative pressure; MO, myocardial oxygen supply:demand index; OPP, ocular perfusion pressure; PSV, peak systolic velocity; RMSDD, root mean square of direct differences of NN intervals; RPP, rate–pressure product; SBP, systolic blood pressure; SDNN, standard deviation of NN intervals; SI, stroke index; SV, systolic velocity; TPR, total peripheral resistance; ${\dot{V}}_{O_{2}}$, oxygen consumption.

^a^Change per 10 mmHg increase in LBNP strength (more negative).

^b^Change from females to males.

^c^Change from 0° supine to 15° head‐down tilt.

^d^Change from left side to right side.

^e^Interaction terms or distributional parameters.

^f^SV interaction: Pressure×Sex.

^g^ ${\dot{V}}_{O_{2}}$ interaction: Sex×Position.

^h^IJVP distributional parameter (log(σ)∼Pressure): $\zeta_{\mathrm{LBNP}}$, such that $\sigma =\sigma_{0}e^{\left(\zeta_{\mathrm{LBNP}}\times Pressure\right)}$.

^i^First intercept gives base log odds of grade 1 vs. grade 2 and 3 flow. Second intercept gives base log odds of grade 1 and 2 vs. grade 3 flow.

^j^EDV interaction 1: Pressure×Sex.

^k^EDV interaction 2: Sex×Position.

^l^EDV interaction 3: Sex×Side.

**TABLE 5:** Summary of main effects in the multivariate dose–response model for all dependent variables considered.

Parameter	$\beta_{\mathrm{LBNP}}$		$\beta_{\mathrm{sex}}$		$\beta_{\mathrm{pos}}$		$\beta_{\mathrm{side}}$	
	pd	${\%}_{\mathrm{ROPE}}$	pd	${\%}_{\mathrm{ROPE}}$	pd	${\%}_{\mathrm{ROPE}}$	pd	${\%}_{\mathrm{ROPE}}$
HR (%)	100	0	72.16	21.91	100	0	–	–
SV (%)	100	0	100	0.01	100	0	–	–
CO (%)	100	0	98.50	3.88	100	0	–	–
${\dot{V}}_{O_{2}}$ (%)	100	0.01	99.58	0.90	99.98	11.57	–	–
SBP (%)	100	4.22	96.61	5.29	63.06	74.23	–	–
DBP (%)	84.01	99.67	87.17	13.44	66.35	69.98	–	–
RPP (%)	100	0	67.61	23.35	99.76	12.14	–	–
MO (%)	100	0	70.49	23.70	99.72	12.54	–	–
TPR (%)	100	0	93.55	10.08	100	0.01	–	–
SI (%)	100	0	94.51	11.47	100	0	–	–
CI (%)	100	0	70.97	31.80	100	0	–	–
SDNN (%)	61.71	100	95.38	5.91	100	0.22	–	–
HRVTi (%)	67.38	99.98	87.65	12.27	99.39	10.39	–	–
RMSDD (%)	100	0.52	75.12	20.49	100	0.15	–	–
BRS (%)	100	21.60	64.48	22.27	100	0.11	–	–
IOP (%)	100	0	58.00	17.06	100	0	51.31	4.49
OPP (%)	93.26	99.49	68.26	21.30	99.45	9.50	51.41	4.33
*A*_IJV_ (%)	100	0	92.05	9.29	100	0	50.84	4.37
IJVP (%)	100	0	89.12	19.17	100	0	51.94	4.53
IJVF (%)	100	0	60.02	16.65	100	0	50.13	14.67
*A*_CCA_ (%)	53.45	100	65.50	15.75	100	0	51.11	4.42
PSV (%)	100	0.01	94.80	7.02	84.87	57.37	53.43	4.41
EDV (%)	84.73	99.26	54.72	8.48	58.36	67.26	50.95	4.54

*Note*: Evidence for existence of effects is presented as probability of direction (pd, where a higher percentage indicates more evidence for a consistent effect in a particular direction). Evidence for significance of effects is presented as a percentage of the full posterior distribution in the region of practical equivalence (${\%}_{ROPE},$where <5% can be interpreted as strong evidence for an effect). See Section 2.5 for details on the pd and ROPE range.

Abbreviations: *A*_CCA_, common carotid artery area; *A*_IJV_, internal jugular vein cross‐sectional area; BRS, baroreflex sensitivity; CI, cardiac index; CO, cardiac output; DBP, diastolic blood pressure; EDV, end‐diastolic velocity; HR, heart rate; HRVTi, heart rate variability triangular index; IJVP, internal jugular vein pressure; IOP, intraocular pressure; MO, myocardial oxygen supply:demand index; OPP, ocular perfusion pressure; PSV, peak systolic velocity; RMSDD, root mean square of direct differences of NN intervals; RPP, rate–pressure product; SBP, systolic blood pressure; SDNN, standard deviation of NN intervals; SI, stroke index; SV, systolic velocity; TPR, total peripheral resistance; ${\dot{V}}_{O_{2}}$, oxygen consumption.

#### 3.2.1 Pressure effect

**FIGURE 6:**
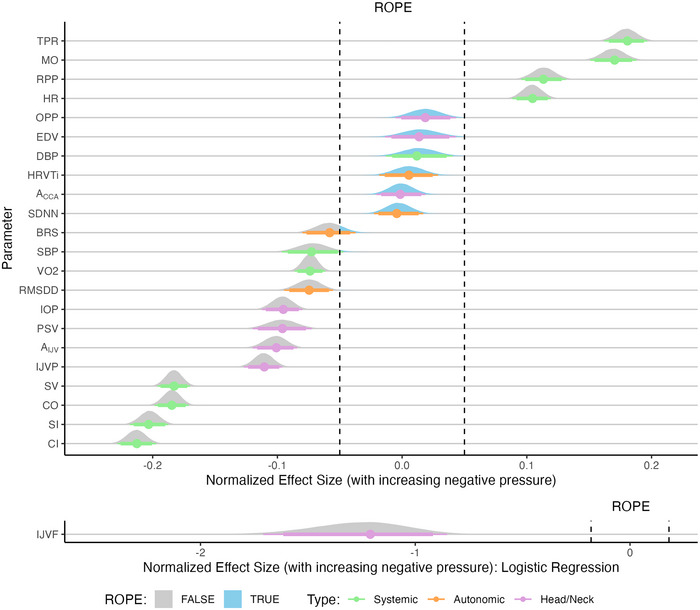
Normalized effect size responses of systemic (green), autonomic (orange) and head/neck (purple) variables to the main effect LBNP Pressure. Variables are ordered from the largest positive effect size at the top of the figure to the largest negative effect size at the bottom. Data are presented as the posterior distributions from the Bayesian multivariate regression model. Distributions are coloured grey when located outside the ROPE and blue when located inside the ROPE. Points and error bars underneath the distributions represent the maximum a posteriori estimate along with the 89% (thick) and 95% (thin) highest density intervals. IJVF is presented separately below, because the ROPE is defined differently for a logistic regression model (for details, see Section 2.5). Abbreviations: IJVP, internal jugular vein pressure; LBNP, lower‐body negative pressure; ROPE, region of practical equivalence.

Figure 6 presents the normalized effect size responses of all variables considered with respect to the main effect Pressure. The variables are ordered from the largest positive effect size at the top of the figure to the largest negative effect size at the bottom. In addition, IJVF is presented separately owing to the differing ROPE range. A positive effect size in a variable indicates an increase in that variable with increasing (i.e., more negative) LBNP. Tables 4 and 5 present the fitted parameters from the dose–response curves and the main effects pd and ${\%}_{ROPE}$, respectively.

Of the four main effects considered, the effect of LBNP Pressure presents the strongest evidence (i.e., narrowest posterior distributions), with the majority of posterior distributions of parameters situated either fully inside or outside the ROPE. The systemic haemodynamic variables are generally those that are most influenced by LBNP Pressure, with a larger relative effect size (either positive or negative) than the autonomic or cephalad variables. In particular, we observe a decrease in SV/CO (and their indexed equivalents) with increasing negative pressure, in addition to the corresponding rise in TPR and MO. In contrast, there is strong evidence in favour of no effect of LBNP Pressure on six variables (*A*_CCA_, SDNN, HRVTi, DBP, OPP, and EDV). These results suggest that: (1) overall HR variability is not influenced by LBNP; and (2) most LBNP‐related effects are related to the systolic (${\%}_{ROPE}=4.22\%$), as opposed to the diastolic (${\%}_{ROPE}=99.67\%$), part of the blood pressure waveform. In addition, related to the head/neck haemodynamics, it is insightful that there is strong evidence for a significant effect of LBNP on IOP, *A*_IJV,_ and IJVP (${\%}_{ROPE}$ = 0% for all three) of approximately similar relative magnitude, yet no effect on OPP (${\%}_{ROPE}$= 99.49%) (Hall & Whittle, 2024). This has potential implications for the use of LBNP as a SANS countermeasure, as discussed in Section 4.

Of the three groups (systemic haemodynamics, autonomic response, and head/neck), the autonomic variables are the least affected by LBNP, although there is still strong evidence of a decrease in parasympathetic activity (decrease in RMSDD: ${\%}_{ROPE}$ = 0.52%). Finally, there is a clear effect of LBNP on the IJVF flow pattern (${\%}_{ROPE}$ = 0%), with the relative log odds of a higher grade (2 or 3) flow decreasing by 1.08 (89% CrI: 0.44 to 2.52) with each 10 mmHg increase in LBNP strength.

#### 3.2.2 Sex effect

**FIGURE 7:**
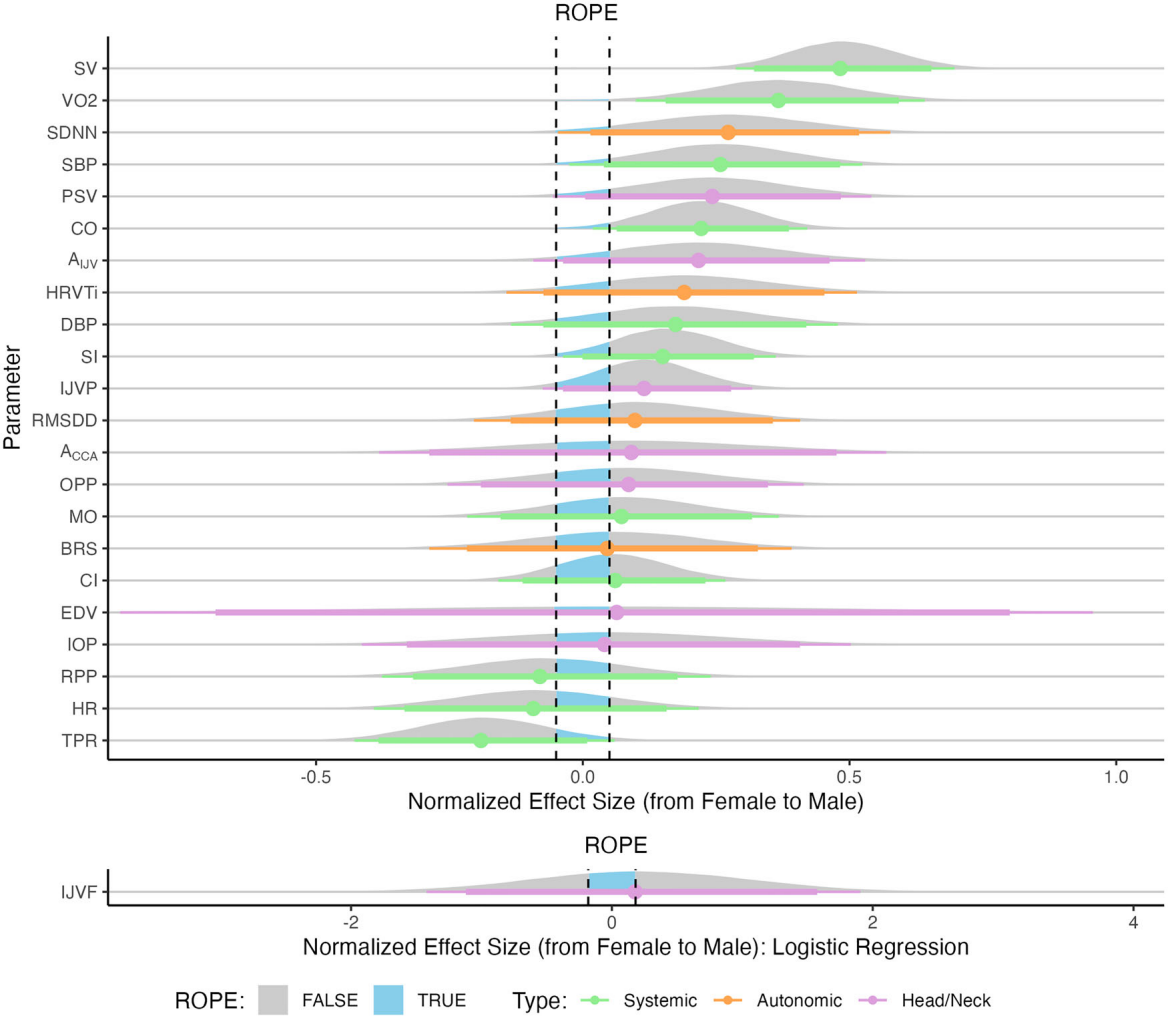
Normalized effect size responses of systemic (green), autonomic (orange) and head/neck (purple) variables to the main effect Sex (male or female). A positive effect size represents an increase in male subjects with respect to female subjects. Variables are ordered from the largest positive effect size at the top of the figure to the largest negative effect size at the bottom. Data are presented as the posterior distributions from the Bayesian multivariate regression model. Distributions are coloured grey when located outside the ROPE and blue when located inside the ROPE. Points and error bars underneath the distributions represent the maximum a posteriori estimate along with the 89% (thick) and 95% (thin) highest density intervals. IJVF is presented separately below, because the ROPE is defined differently for a logistic regression model (for details, see Section 2.5). Abbreviations: IJVP, internal jugular vein pressure; ROPE, region of practical equivalence.

Figure 7 presents the normalized effect size responses of all variables considered with respect to the main effect Sex. The variables are ordered from the largest positive effect size at the top of the figure to the largest negative effect size at the bottom. In addition, IJVF is presented separately owing to the differing ROPE range. Due to the contrast coding in the model, a positive effect size in a variable represents an increase in that variable in male subjects compared with female subjects. Tables 4 and 5 present the fitted parameters from the dose–response curves and the main effects pd and ${\%}_{ROPE}$, respectively.

In contrast to the effect of Pressure, the posteriors associated with the Sex effect are much wider. This is attributable to the fact that the magnitude of any sex effect between males and females is often overshadowed by the natural intersubject variability found across all subjects. We found strong evidence of significant effects of Sex in only three variables (SV, ${\%}_{ROPE}=0.01\%$; ${\dot{V}}_{O_{2}}$, ${\%}_{ROPE}=0.90\%$; and CO, ${\%}_{ROPE}=3.88\%$). With respect to the indexed variables, there is minimal evidence of a sex effect in CI (pd=70.97%, ${\%}_{ROPE}=31.80\%$) and marginal evidence of a sex effect in SI (pd=94.51%, ${\%}_{ROPE}=11.47\%$). The presence of a significant effect in the absolute variables (CO and SV) and the lack of a significant effect in the indexed variables (and elsewhere) would appear to indicate that differences between males and females are principally driven by anthropometric differences (i.e., on average males are larger, with a higher total blood volume and a larger SV).

In four additional variables, there is moderate evidence of the presence of a sex effect, even if it is not necessarily of a significant magnitude. This is evidenced by variables with a pd of >90% and a ${\%}_{ROPE}$ of <10% (in simple terms, the majority of the posterior distribution is away from zero). These variables are SDNN (pd=95.38%, ${\%}_{ROPE}=5.91\%$), PSV (pd=94.80%, ${\%}_{ROPE}=7.02\%$), SBP (pd=96.61%, ${\%}_{ROPE}=5.29\%$) and *A*_IJV_ (pd=92.05%, ${\%}_{ROPE}=9.29\%$). The fact that SDNN appears larger in males (by, on average, 12.2 ms, 89% CrI: 0.6 to 23.2 ms) is likely to be explained by hormonal effects on the autonomic nervous system (Dart et al., 2002). We do not, however, find a corresponding reduction in RMSDD in males, which would be indicative of higher parasympathetic activity in females.

In summary, a comparison between absolute variables (CO and SV) and indexed variables (CI and SI) suggests that sex differences in the data appear to be driven principally by anthropometric variation between males and females. However, there is some evidence of increased sympathetic activity in male subjects.

#### 3.2.3 Position effect

**FIGURE 8:**
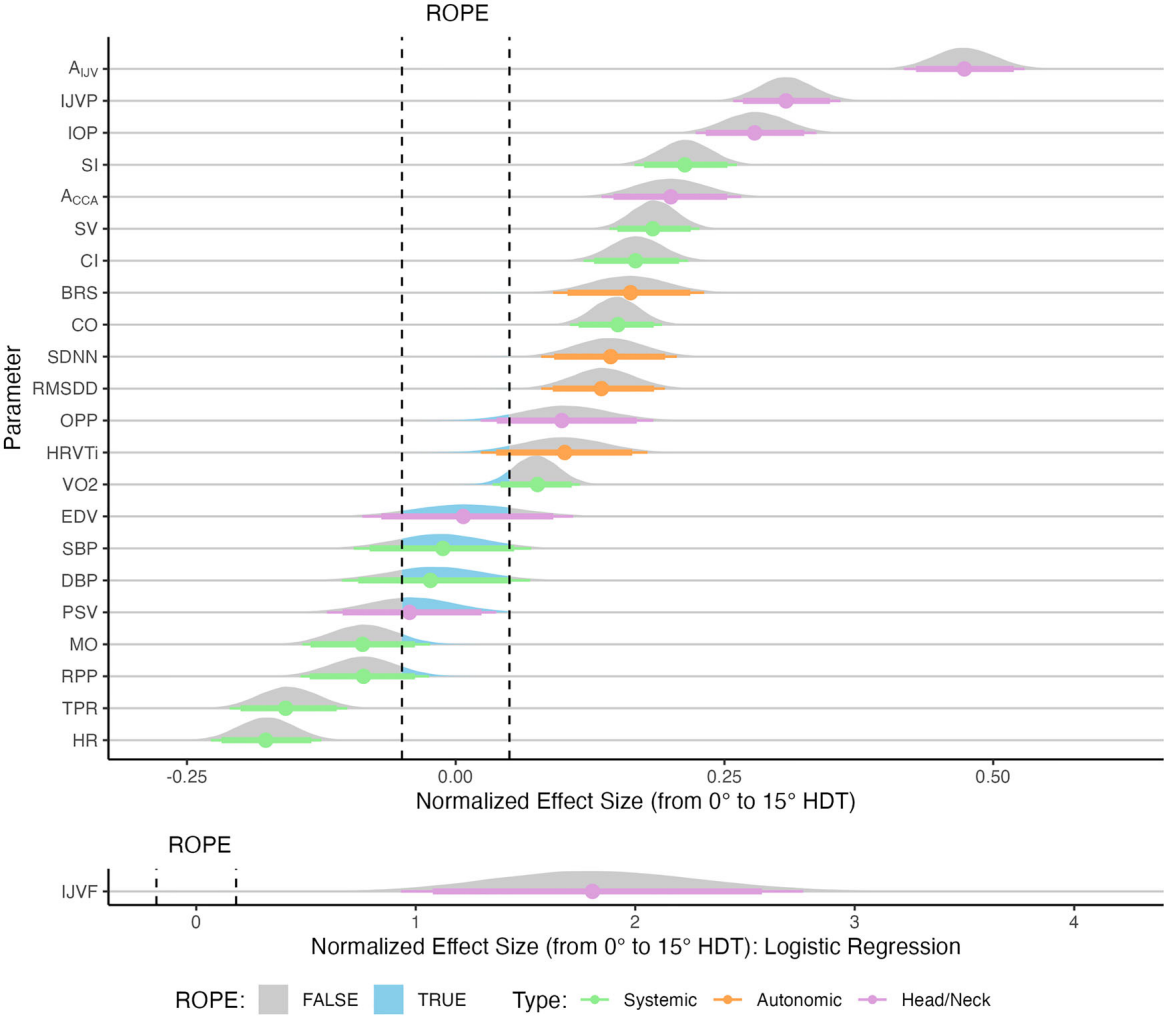
Normalized effect size responses of systemic (green), autonomic (orange) and head/neck (purple) variables to the main effect Position (0° supine or 15° HDT). A positive effect size represents an increase in 15° HDT with respect to 0° supine. Variables are ordered from the largest positive effect size at the top of the figure to the largest negative effect size at the bottom. Data are presented as the posterior distributions from the Bayesian multivariate regression model. Distributions are coloured grey when outside the ROPE and blue inside the ROPE. Points and error bars underneath the distributions represent the maximum a posteriori estimate along with the 89% (thick) and 95% (thin) highest density intervals. IJVF is presented separately below, because the ROPE is defined differently for a logistic regression model (for details, see Section 2.5). Abbreviations: IJVP, internal jugular vein pressure; ROPE, region of practical equivalence.

The main effect of position is insightful because it relates to the characterization of LBNP responses in the presence of a cephalad fluid shift. Figure 8 presents the normalized effect size responses of all variables considered with respect to the main effect Position. The variables are ordered from the largest positive effect size at the top of the figure to the largest negative effect size at the bottom. In addition, IJVF is presented separately owing to the differing ROPE range. Based on the coding scheme that was used to capture the main effect Position in the model, a positive effect size represents an increase from 0° supine to 15° HDT. Tables 4 and 5 present the fitted parameters from the dose–response curves and the main effects pd and ${\%}_{ROPE}$, respectively.

A significant effect is seen in 6 of the 11 systemic haemodynamic parameters, three of the autonomic parameters and five of the eight head/neck parameters. The largest effect sizes are found in the jugular vein response (area, pressure, and flow). This is congruent with our previous studies (Whittle & Diaz‐Artiles, 2023), which demonstrated the strong gravitational dependence of the jugular vein. In contrast to Whittle and Diaz‐Artiles (2023), we also find a significant effect of Position on *A*_CCA_ (pd=100%, ${\%}_{ROPE}=0\%$), although it should be noted that in the present study, we are only considering a unique tilt angle. We also note significant increases with 15° HDT in SV (pd=100%, ${\%}_{ROPE}=0\%$), CO (pd=100%, ${\%}_{ROPE}=0\%$) (and their indexed equivalents), IOP (pd=100%, ${\%}_{ROPE}=0\%$), BRS (pd=100%, ${\%}_{ROPE}=0.11\%$), SDNN (pd=100%, ${\%}_{ROPE}=0.22\%$) and RMSDD (pd=100%, ${\%}_{ROPE}=0.15\%$), along with significant decreases in HR (pd=100%, ${\%}_{ROPE}=0\%$) and TPR (pd=100%, ${\%}_{ROPE}=0.01\%$).

The RMSDD and BRS responses, combined with the decrease in HR, indicate that the autonomic response is activated by the cephalad fluid shift, manifested principally by an increase in vagal activity, lowering HR. This is combined with the reduced TPR promoting venous return, leading to increased SV and CO through the Frank–Starling mechanism (Whittle & Diaz‐Artiles, 2021).

Finally, we note an increase in OPP in 15° HDT (pd=99.45%, ${\%}_{ROPE}=9.50\%$), which is significant in direction and approaching significance in magnitude. This is congruent with our previous studies demonstrating an increase in OPP in HDT (Petersen et al., 2022). Analysis of the relative magnitudes of the effect sizes indicates that the increase in OPP is blunted by the constancy of blood pressure, both SBP and DBP, which present evidence in favour of the null hypothesis of no effect of position (SBP, pd=63.06%, ${\%}_{ROPE}=74.23\%$; and DBP, pd=66.35%, ${\%}_{ROPE}=69.98\%$).

#### 3.2.4 Side effect

**FIGURE 9:**
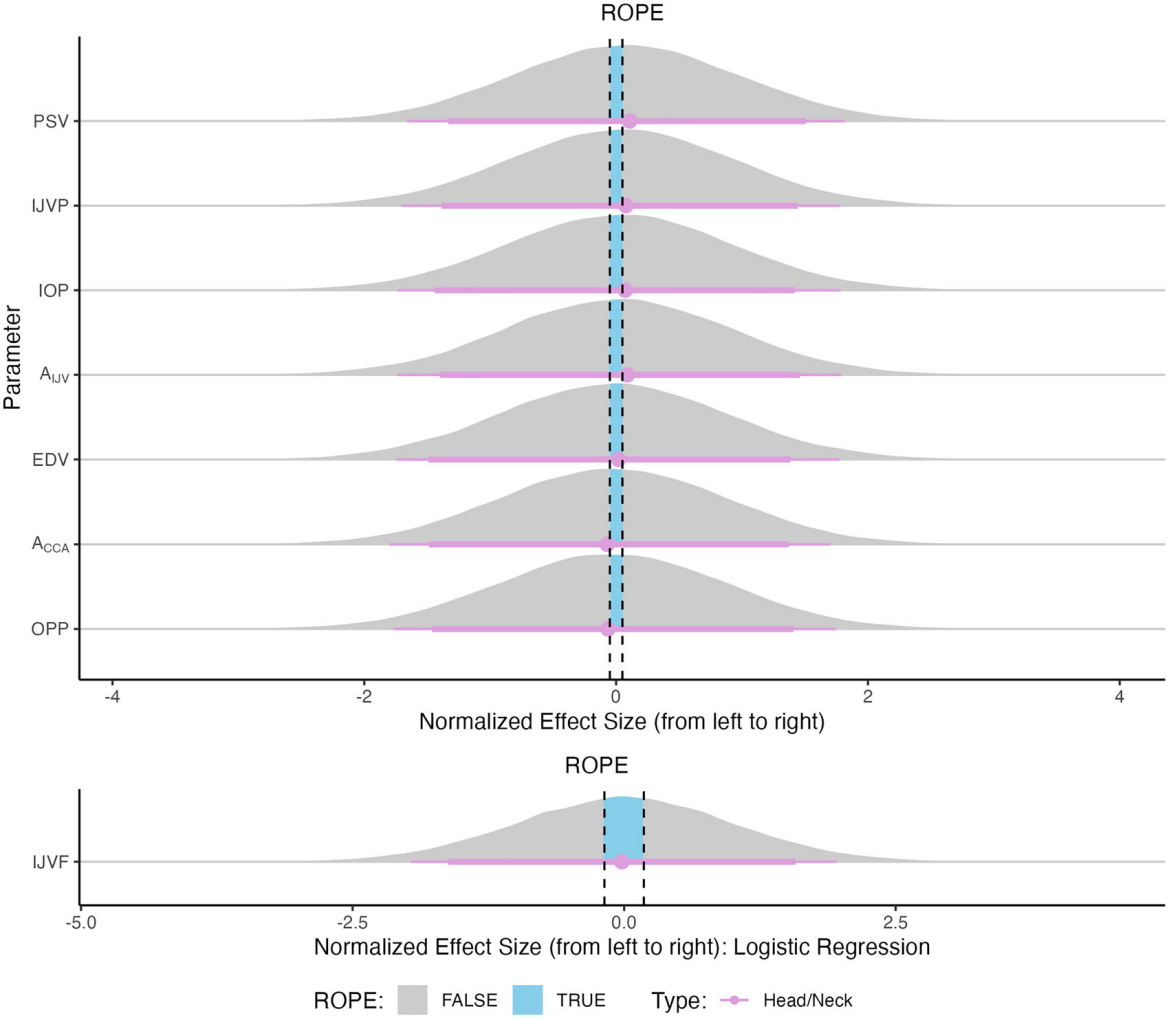
Normalized effect size responses of head/neck (purple) variables to the main effect Side (left or right). A positive effect size represents an increase in the right side with respect to the left side. Variables are ordered from the largest positive effect size at the top of the figure to the largest negative effect size at the bottom. Data are presented as the posterior distributions from the Bayesian multivariate regression model. Distributions are coloured grey when outside the ROPE and blue inside the ROPE. Points and error bars underneath the distributions represent the maximum a posteriori estimate along with the 89% (thick) and 95% (thin) highest density intervals. IJVF is presented separately below, because the ROPE is defined differently for a logistic regression model (for details, see Section 2.5). Abbreviations: IJVP, internal jugular vein pressure; ROPE, region of practical equivalence.

Figure 9 presents the normalized effect size responses of the head/neck variables with respect to the main effect Side. The variables are ordered from the largest positive effect size at the top of the figure to the largest negative effect size at the bottom. In addition, IJVF is presented separately owing to the differing ROPE range. Owing to the contrast coding in the model, a positive effect size represents an increase in the right side with respect to the left side. Tables 4 and 5 present the fitted parameters from the dose–response curves and the main effects pd and ${\%}_{ROPE}$, respectively.

In all eight variables considered, we did not find an effect of Side. For all variables except IJVF, both the ${\%}_{ROPE}$ is small (<5%) and the pd is ∼50%–54%. This is owing to the broad posterior distributions roughly centered around zero. Thus, there is no evidence of an effect size in any particular direction. This is in contrast to our results from previous tilt experiments (Petersen et al., 2022; Whittle & Diaz‐Artiles, 2023; Whittle et al., 2022), in which we found differences in size between the left and right IJVs. It would appear that this side effect is present only in the amplified expansion of the IJV in large HDTs. Note that, in the Bayesian framework, our results suggest that there is no significant evidence either in favour of the null hypothesis (no effect) or the alternative (an existing effect). Aside from our findings on *A*_IJV_ in tilt discussed above, the rest of the results presented here are congruent with our previous results, such that we found no effect of side on IOP, OPP, or IJVP.

#### 3.2.5 Internal jugular vein flow

**FIGURE 10:**
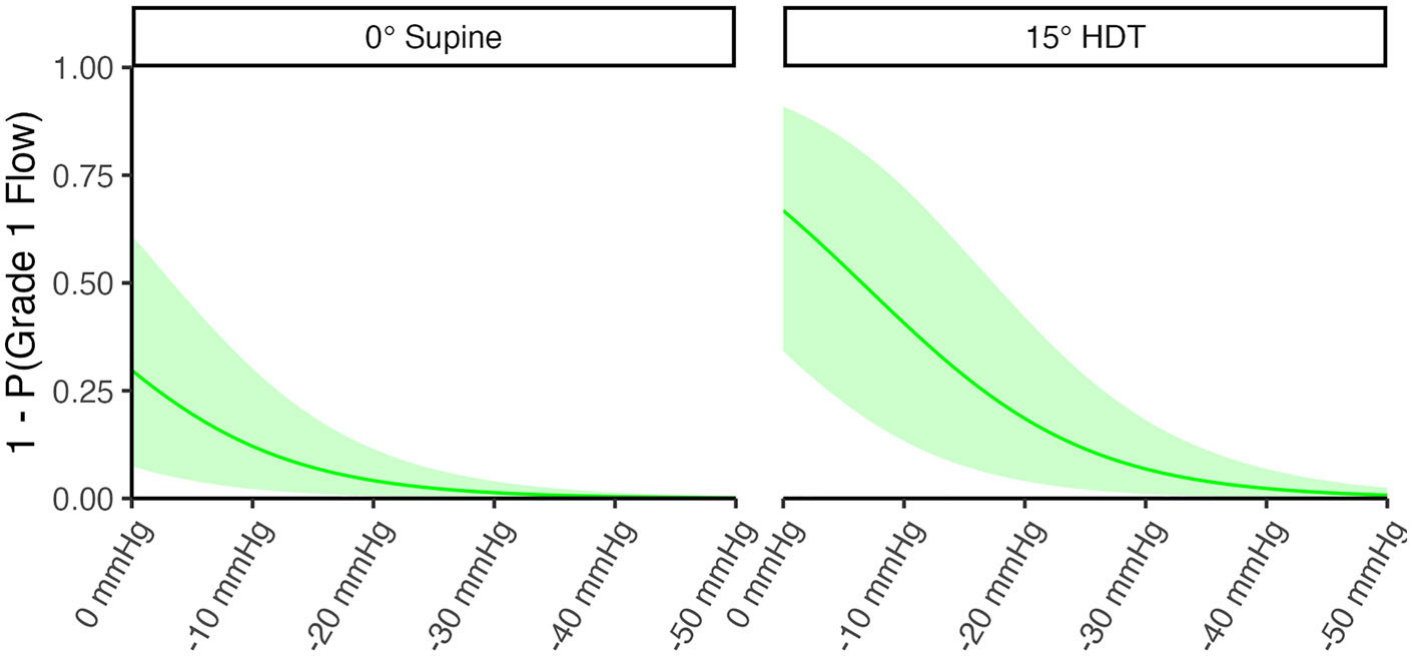
Dose–response curve for internal jugular vein blood flow velocity waveform pattern. The y‐axis represents the probability of having a higher than grade 1 flow (i.e., grade 2 or grade 3 flow). Dose–response represents the fitted posterior draws from the multivariate regression model. Sex and Side effects are pooled. Position (0° supine or 15° HDT) is faceted. Dose–response is presented as the maximum a posteriori estimate ± 89% CrI. Abbreviations: CrI, credible intervals; HDT, head‐down tilt.

The majority of the variables are explained by linear models, whose interpretation is relatively straightforward. In contrast, the dose–response curve for the IJV blood flow velocity waveform pattern is based on an ordinal logistic regression, with a slightly more cryptic interpretation. Thus, it is insightful to discuss this dose–response in detail. Figure 10 presents the IJV flow pattern dose–response curve. Based on the effect sizes in Figures 6, 7, 8, 9 (where there was evidence of Pressure and Position effects, but no evidence of Sex or Side effects), we have grouped males and females and left and right sides together. Although the response is ordinal, the figure shows the latent variable given by the logit function (DeMaris, 1995). Owing to only four instances of grade 3 flow occurring (Figure 5), we have grouped the probabilities such that the *y*‐axis in the curve represents the probability of ‘greater than grade 1 flow’ (i.e., grade 2 or grade 3 flow).

In 0° supine, the probability of greater than grade 1 flow is 29.8% (89% CrI: 7.5% to 60.9%) at 0 mmHg. This is reduced by LBNP to 12.1% (89% CrI: 2.2% to 30.1%), 4.1% (89% CrI: 0.6% to 11.4%), 1.3% (89% CrI: 0.1% to 4.0%), 0.4% (89% CrI: 0.0% to 1.4%) and 0.1% (89% CrI: 0.0% to 0.4%) at −10, −20, −30, −40 and −50 mmHg, respectively. In 15° HDT, there is an increased probability of grade 2 or higher flow of 66.8% (89% CrI: 34.2% to 90.9%) at 0 mmHg. This is reduced by LBNP to 40.7% (89% CrI: 13.4% to 72.2%), 18.5% (89% CrI: 4.0% to 42.0%), 6.8% (89% CrI: 1.0% to 18.2%), 2.3% (89% CrI: 0.2% to 6.8%) and 0.8% (89% CrI: 0.1% to 2.5%) at −10, −20, −30, −40 and −50 mmHg, respectively.

The data highlight that LBNP effectively increases blood flow in the IJVs, particularly in 15° HDT. The implications of these results will be discussed further in Section 4.

#### 3.2.6 Multivariate relationships

**FIGURE 11:**
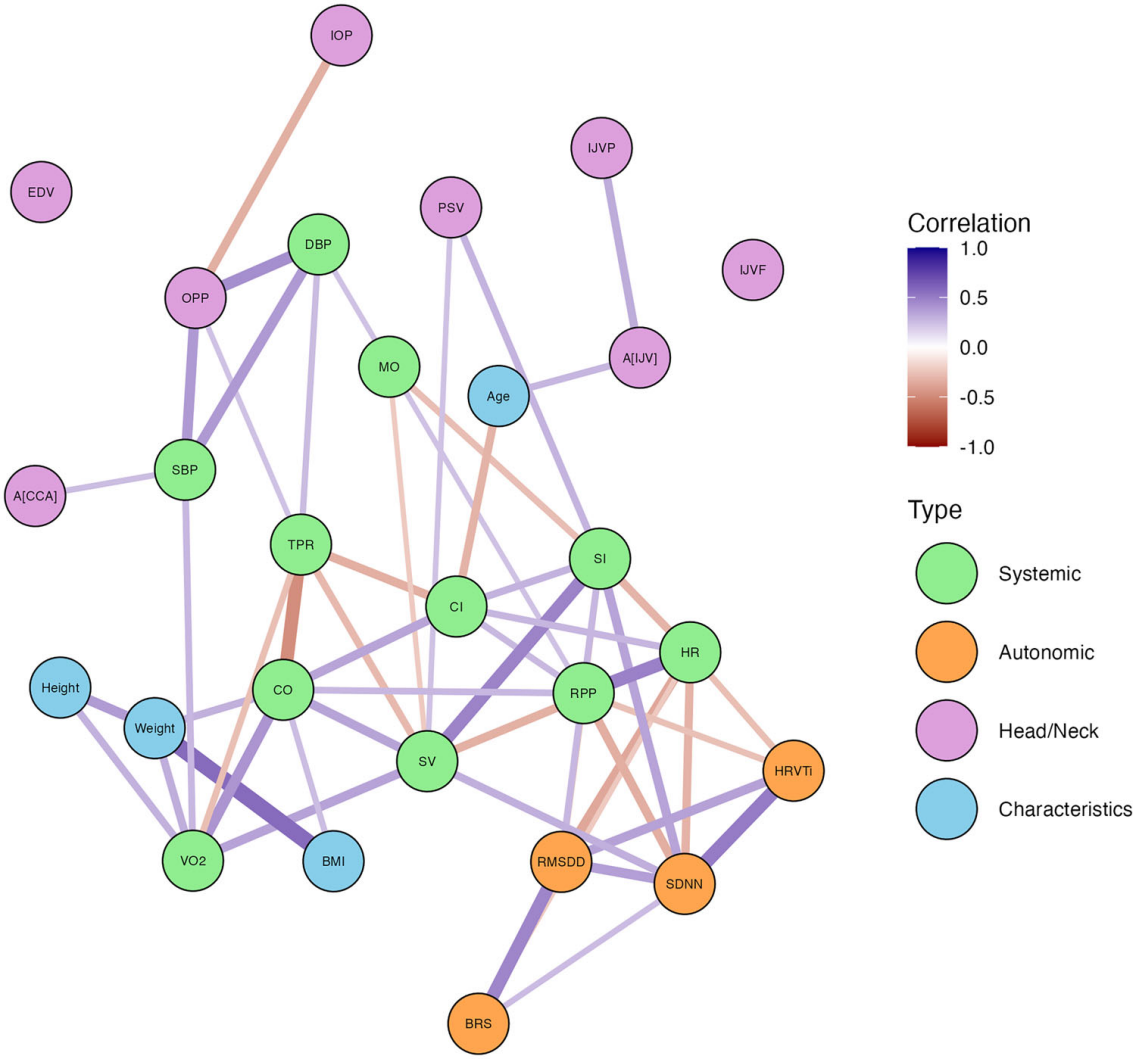
Graph structure representing the multivariate relationships amongst all the measured variables (green, systemic; orange, autonomic; purple, head/neck; blue, subject characteristics). The direction of the correlations (positive or negative) is represented by the colour of the edges, and the strength of the correlations (the maximum a posteriori estimate) is represented by the thickness of the edges. Only significant correlations are shown (see main text for details).

Figure 11 presents the multivariate relationships amongst all the variables considered, including the subject characteristics (Age, Height, Weight, and BMI). Correlations are displayed only when there is significant evidence of an effect (in the Bayesian formulation, this is when the full posterior distribution does not encompass zero). In comparison to correlation in a frequentist framework, the maximum a posteriori estimate is analogous to the r‐value, and the significance, i.e., whether the posterior distribution encompasses zero, is analogous to the p‐value.The inclusion of the subject characteristics also provides insight into any relationships between the variables driven by anthropometric considerations such as height or weight.

Broadly, the graph structure appears to form two connected groups. All the autonomic parameters (in orange) are strongly connected to one another, forming one group. Likewise, the systemic haemodynamic parameters (in green) form a second connected group structure. The HR is linked to the autonomic parameters through HR variability (associated with both SDNN and HRVTi) and parasympathetic activity (RMSDD), whereby higher HR is associated with lower variability and lower vagal activity. Likewise, increased heart rate variability is associated with increased SV and reduced RPP.

The OPP is associated with both blood pressure numerics (SBP and DBP) and IOP, but increased OPP is also associated with increased TPR. The relationship with the subject characteristics is also insightful. In contrast to Buckey et al. (2022, 2018), we do not find any association between IOP and body weight or BMI. However, greater height and weight are associated with increased ${\dot{V}}_{O_{2}}$. More interestingly, we see that age is positively correlated with IJV cross‐sectional area, *A*_IJV_ (and, by extension, pressure, IJVP) and negatively correlated with CI. Finally, carotid haemodynamics are associated with SBP (correlated with *A*_CCA_) and SV (correlated with PSV). There is no significant relationship between EDV or IJVF and any of the other metrics.

## 4 DISCUSSION

In this study, we investigated the acute effects of LBNP on the cardiovascular system. To our knowledge, this is the most comprehensive analysis of cardiovascular haemodynamics, autonomic, and cephalad response to LBNP to date. Our main findings suggest that: (1) there is a varying magnitude of the normalized effect sizes of responses to LBNP in different cardiovascular variables; (2) sex differences exist between the male and female response, but these appear to be driven principally by anthropometric considerations; (3) concerning head/neck variables (i.e., CCAs, IJVs, and eyes), there is no evidence of a difference between the response in the left and right sides to LBNP (within the experimental conditions considered); and (4) there is an underlying multivariate structure, with associations connecting all but two (EDV and IJVF) of the variables considered, including cardiovascular variables, in addition to subject characteristics such as age, height, weight, and BMI.

Multiple studies have investigated the effects of LBNP on cardiovascular haemodynamics; many of these are summarized in a comprehensive review article by Goswami et al. (2019). In a classic study by Blomqvist and Stone (2011), the systemic haemodynamic responses to graded LBNP up to −40 mmHg were shown to be linear for TPR, HR, SV, CO, and MAP. Data from their study were compiled by Goswami et al. (2019). Our study, and in particular Figure 6, extends this work and increases the number of variables considered. Our findings agree with the authors regarding the relative magnitudes of the effects of TPR (large positive), HR (small positive), and CO and SV (large negative). We also find no decrease in DBP and a small decrease in SBP, which correspond well to the minimal decrease in MAP found by Goswami et al. (2019). In Figure 6 we add the effect sizes of 17 new variables and present the relative magnitudes of the LBNP‐induced changes. Of note, we find that, although the TPR and SV/CO changes are the largest positive and negative effects, respectively, there are smaller, still significant, negative effects surrounding the jugular vein (*A*_IJV_, IJVP and IJVF), IOP, and ${\dot{V}}_{O_{2}}$, in addition to positive effects on myocardial oxygen supply:demand index (MO) and RPP.

The autonomic response to LBNP is mediated principally through the reduction in central blood volume, lowering systemic flow and perfusion pressure, leading to stimulation of the arterial baroreflex (Goswami et al., 2019; Roddie & Shepherd, 1958; Rowell, 1993; Victor & Mark, 1985). Convertino et al. (2012) investigated the effect of LBNP on BRS, finding that BRS decreased from 15 ± 1 to 7 ± 1 ms/mmHg at presyncope in low‐tolerance subjects and from 17 ± 2 to 4 ± 0 ms/mmHg in high‐tolerance subjects. This matches well with our data, which show a reduction of BRS from 13.8 ± 1.1 ms/mmHg at 0 mmHg to 9.2 ± 0.7 ms/mmHg at −50 mmHg (average of both sexes and both positions). We did not deliberately take our subjects to the point of presyncope, but the decreasing trend is anticipated to continue to that point. This decrease in BRS, combined with the reduction in time‐derived heart rate variability metrics (i.e., RMSDD), is indicative of progressive vagal withdrawal (Cooke & Convertino, 2005; Cooke et al., 2008; Pomeranz et al., 1985). Experiments using cholinergic blockade demonstrate the importance of both arms of the autonomic response (vagal withdrawal and sympathetic activation) to mediate cardiac function in LBNP (Convertino, 2014; Convertino & Sather, 2000). In addition, the sympathetic response is also important for mediating vascular smooth muscle constriction in response to the reduction in central blood volume (Convertino et al., 2012).

Regarding sex differences, Convertino (1998) investigated differences in autonomic function related to blood pressure regulation. Our results are congruent with those of Convertino, who noted higher HR in female subjects, combined with a lower SV during LBNP. Convertino took all subjects to presyncope and noted a lower tolerance in females. Although we did not deliberately take our subjects to presyncope, the fact that six of the seven subjects who reached presyncope whilst supine in the 0 to −50 mmHg range were female suggests a lower orthostatic tolerance in females. This finding is well supported by multiple studies (Carter et al., 2015; Convertino, 1998; Franke et al., 2003; Goswami et al., 2015; Hinojosa‐Laborde et al., 2015; White et al., 1996). Convertino also derived the effect size of the LBNP response in males and females with regard to HR, SV, CO, MAP and TPR, noting that the difference in slope between males and females was non‐significant (p>0.05) in all cases except for TPR (p=0.0002). On the autonomic side, Convertino measured the BRS, noting that the response was 1.32 ms/mmHg lower in females (p=0.047). We found that BRS was potentially higher in males by 0.6 ms/mmHg (89% CrI: −3.1 to 4.5 ms/mmHg), although the difference was far from significant and outweighed by intersubject variability (pd=64.48%, ${\%}_{ROPE}=22.27\%$). Other studies have found potential differences in the autonomic response between men and women. Frey et al. (1986) found that women have a more dominant vagal response, whereas Kelly et al. (2004) suggested that men primarily demonstrate a greater sympathetic response. In addition, Frey and Hoffler (1988) also found that men exhibited a larger increase in TPR. By considering Figure 7, we find some trending evidence of a higher vagal response in males based on RMSDD (pd=75.12%, ${\%}_{ROPE}=20.49\%$). Further study is required to investigate these discrepancies in the sex‐driven autonomic response. More recently, Patterson et al. (2022) measured *A*_IJV_ differences between males and females during LBNP exposure in the range from 0 to −40 mmHg. They noted a significant effect of sex (p<0.001) and LBNP (p<0.001), but no significant interaction (p=0.066), with *A*_IJV_ being larger in female subjects at 0 , −20 and −30 mmHg. In contrast, we found a significant effect of LBNP of −0.84 mm^2^/mmHg (89% CrI: −0.97 to −0.73 mm^2^/mmHg) but no significant effect of sex. In fact, in our study we found trending evidence of a higher *A*_IJV_ in males by 18.1 mm (Hargens & Richardson, 2009) (89% CrI: −3.1 to 38.7 mm (Hargens & Richardson, 2009); pd=92.05%, ${\%}_{ROPE}=9.29\%$). Our results match previous studies, such as those by Jeon et al. (2020) and Magnano et al. (2016), who found no difference in *A*_IJV_ between males and females.

The article by Magnano et al. (2016) is particularly interesting in that they note a positive association between *A*_IJV_ and age in >1000 subjects. Using a totally different methodology, we also discovered this association, captured in Figure 11. This finding lends support both to their conclusions and to our Bayesian modelling workflow. Magnano et al. (2016) hypothesize that the increased *A*_IJV_ is linked to inhibited central venous drainage as a result of raised intra‐abdominal pressure with increased BMI, which trends to be higher in older individuals. In contrast, although admittedly in a far smaller study, we find no direct link between BMI (or weight) and *A*_IJV_. This suggests that the association might be related to other factors apart from BMI. Magnano et al. (2016) posit the role of endothelial progenitor cells in the vascular remodelling process, noting sex differences related to pregnancy hormones (Robb et al., 2007). Given evidence that endothelial progenitor cells decrease with increasing age (Yang et al., 2018), it could be possible that endothelial progenitor cells play a role in the age‐related differences in *A*_IJV_. Continuing our discussion of Figure 11, we also note the negative association between age and CI, which is supported by a number of studies (Cioccari et al., 2019; Katori, 1979). Furthermore, we also find an association between OPP and TPR. It is difficult to find evidence in the literature for similar relationships, although Fındıkoğlu et al. (2015) noted a decrease in OPP in 34 subjects after hot‐water immersion, which was also matched by a decrease in TPR.

The effect of position is important because it allows us to determine the relative magnitude of the effect of a headward fluid shift induced by HDT (and, by extrapolation, microgravity) in comparison to the LBNP effect. Of the three groups of variables (systemic, autonomic and cephalad), we note that the largest effect size increases occur in the head/neck parameters, including *A*_IJV_, IJVP, IOP and IJVF. Interestingly, in this experiment we also found evidence of an increase in *A*_CCA_, which we did not observe in our previous tilt study (Whittle & Diaz‐Artiles, 2023). It should be noted that the present study examines only a single tilt angle and that compensatory mechanisms might prevent this increase from progressing at more severe HDT angles. Aside from *A*_CCA_, there are no data that contradict our previous experiments, including evidence that blood pressure is maintained in tilt (Petersen et al., 2022; Whittle & Diaz‐Artiles, 2023; Whittle et al., 2022).

We did not find any differences in any of the head/neck variables between the left and right sides. This is supported by our previous work examining eye pressures in a tilt protocol, where results suggest that there is no difference in the pressures in the ocular system between the left and right side (neither in IOP nor in OPP) (Petersen et al., 2022). Furthermore, in the study by Whittle & Diaz‐Artiles (2023), we considered the gravitational effects of the carotid arteries and jugular veins in tilt. In that study, we did not measure PSV or EDV; however, we could anticipate that there is little difference between sides in those variables because the CCA branch is located immediately superior to the ascending aorta, such that arterial flow in both sides of the CCA is still approximately equivalent to aortic flow velocity prior to bifurcation. The only difference found between the left and right sides in the tilt study occurred in the *A*_IJV_ (Whittle & Diaz‐Artiles, 2023). We did not find a significant difference in IJVP between the left and right sides, hypothesized as attributable to the fact that both sides sit minimally above central venous pressure.

The main difficulty in detecting significance between the left and right sides is the large intersubject variability. Ogoh et al. (2022) assessed the difference between the left and right side *A*_IJV_ in two conditions: (1) 0° supine with −60 mmHg LBNP; and (2) 60° HUT with no LBNP. This is one of the few studies to have assessed both sides of the jugular veins. Similar to our data, Ogoh et al. (2022) found large variability in their data. With the application of −60 mmHg LBNP, they measured a change in the right *A*_IJV_ of −45% ± 49% and in the left *A*_IJV_ of −49% ± 27% (with respect to the supine position and no LBNP). Here, as with our data, the standard deviation of the measurements is too large to draw significant conclusions. This gives us confidence that indeed, in the variables considered, natural variability between subjects is larger than intrasubject differences between the left and right sides.

### 4.1 Implications for countermeasure design

This study provides new insights into countermeasure design, but it also leads to important questions that warrant future investigation. In particular, related to human spaceflight risks, the three most relevant are: (1) the risk of cardiovascular adaptations contributing to adverse mission performance and health outcomes; (2) the risk of SANS; and (3) the concern of VTE. Each of these risks is considered briefly in the following paragraphs.

#### 4.1.1 Risk of cardiovascular adaptations contributing to adverse mission performance and health outcomes

Given that this study considered only the acute effects of LBNP, it is difficult to make firm recommendations about a long‐term countermeasure for cardiovascular health. We note that the root aetiology of the cardiovascular risk is through fluid shifts leading to: (1) alterations in intravascular volume; (2) changes in cardiac and vascular structure/function; (3) oxidative stress; and (4) inflammation (Antonsen et al., 2022). Reducing the headward fluid shift or at least providing periodic unloading could prevent or reduce the effect of these causal pathways. The open questions remaining are therefore, what are the specific levels and protocols of LBNP that are appropriate to mitigate these risks?

This study provides some insight into these questions. First, we note the well‐established differences in tolerance between males and females. In our study, we found strong evidence of a difference between the male and female response in absolute variables (e.g., SV, CO) but not in indexed variables (e.g., SI, CI). These results suggest that sex differences are driven principally by differences in anthropometry. This places an upper limit on the level of LBNP that could be used in a spaceflight environment, particularly when the astronauts are in a deconditioned state. From our data, for example, a strength of −50 mmHg caused presyncope in more than half of our female subjects. Further analysis might lead to the development of personalized protocols based on anthropometric considerations, rather than a ‘one size fits all’ approach. Second, and most importantly, by considering Figure 6 together with Figure 8, we gain new insight into the relative magnitude of the LBNP Pressure effect compared with a gravitationally induced fluid shift (i.e., Position effect). Thus, we can use these effect sizes and associated models to determine the appropriate level of LBNP needed to bring back any variable of interest to ‘Earth normal’ conditions when subjected to a headward fluid shift. As one example, we note that a 15° HDT increases CO by 0.39 L/min (89% CrI: 0.30 to 0.48 L/min) with respect to supine levels. In contrast, LBNP reduces CO by 0.048 L/min/mmHg (89% CrI 0.045 to 0.050 L/min/mmHg). Thus, it would appear that only −8.1 mmHg of LBNP is required to return CO to its baseline, supine value. As expected, the amount of LBNP required to return to a ‘baseline’ state depends on the variable being considered. For example, for IOP, approximately −28 mmHg of LBNP is required to return IOP to supine levels (when at 15° HDT). Thus, an analysis of which variables are most important to control, combined with an improved understanding of the long‐duration effects, could provide target levels of LBNP for further investigation. It should be noted that there are differences between the physiological response to spaceflight and HDT. In particular, as previously discussed, HDT replicates the fluid shifts but does not remove hydrostatic gradients or alter tissue weight. In addition, the fluid redistribution induced by 15° HDT is most likely to be of a larger magnitude than the fluid redistribution experienced in true microgravity conditions (which are typically simulated using a 6° HDT protocol). Thus, countermeasure development will require further investigation and validation of the resultant protocols in microgravity conditions in addition to terrestrial development.

#### 4.1.2 Risk of SANS

In this study, we find that OPP is not influenced by LBNP in the pressure and position ranges measured. An HDT increases OPP, but there is significant evidence for no effect of LBNP Pressure subsequently to reduce it $(\;{\%}_{ROPE}=99.49\%$). Given the potential relationship between SANS and elevated OPP noted by Petersen et al. (2022) and the symptomatic similarities between terrestrial traumatically elevated OPP and SANS (Petersen et al., 2022; Albano de Guimarães et al., 2021), LBNP could perhaps not be an effective SANS countermeasure. The potential implications of the effects of LBNP on OPP and, more broadly, SANS, are discussed further in a separate publication by Hall and Whittle (2024). The pathoaetiology of SANS is currently unknown, but it is likely to be the result of multiple contributing factors. Related to fluid pressures, although OPP might be important, there are other pressure gradients that are also likely to play a role, including the translaminar pressure gradient (Petersen et al., 2022). An important missing piece of information for determining a more complete haemodynamic environment of the ocular system is a measurement of ICP. Petersen et al. (2019) demonstrated that LBNP can reduce ICP in a study using 10 subjects with either parenchymal ICP sensors or Ommaya reservoirs fitted to the frontal horn of a lateral ventricle. They found that graded LBNP (in the same range as our experiment) reduced ICP from 15 ± 2 (0 mmHg LBNP) to 14 ± 4 (−10 mmHg LBNP), 12 ± 5 (−20 mmHg LBNP), 11 ± 4 (−30 mmHg LBNP), 10 ± 3 (−40 mmHg LBNP) and 9 ± 4 mmHg (−50 mmHg LBNP) (p<0.0001), but that cerebral perfusion pressure ($=\mathrm{MA}P_{\mathrm{mid}-\mathrm{brain}}-\mathrm{ICP}$) was unchanged. It is difficult to obtain non‐invasive measurements of ICP, but there are a number of existing techniques with varying degrees of accuracy that could be leveraged, including CT and MRI, transcranial Doppler, electroencephalography power spectrum analysis and audiological and ophthalmological techniques (Kristiansson et al., 2013). Future work should assess the totality of haemodynamic measurements in the head and eyes, including IOP, OPP, ICP, and cerebral perfusion pressure.

#### 4.1.3 Concern of VTE

Our results quantify the changes in the IJV blood flow velocity waveform pattern as a function of applied LBNP. In Figure 10, we demonstrated that increasing LBNP can decrease the probability of a grade 2 or higher flow. Grade 1 and grade 2 flows are considered normal, whereas the risk of VTE is elevated when attaining grade 3 and grade 4 flows (Marshall‐Goebel et al., 2019). Given that this was an acute study, we observed only four instances of grade 3 flow in three subjects (two male and one female). In contrast, Marshall‐Goebel et al. (2019) observed grade 3 or higher flow in 7 of 11 subjects in flight. Thus, our data mainly demonstrate the ability of LBNP to change grade 2 flow to grade 1 flow. In order to gain a better understanding of the suitability of LBNP as a countermeasure to mitigate the concern of VTE, future work should focus on long‐duration head‐down tilt bed rest studies and especially spaceflight studies, where grade 3 or higher flow is more likely to occur.

### 4.2 Discussion of the Bayesian workflow methodology

An important contribution of this research effort is the Bayesian workflow used to construct LBNP dose–response curves. We believe that this methodology, which moves away from more common and traditional null hypothesis significance testing, is better suited to the analysis of both ground‐based and spaceflight studies of physiological response, which are often plagued by a smaller subject pool. In particular, by removing the reliance on a single value, for example, p≤0.05, to make binary decisions about a null hypothesis, we are able to gather evidence both in favour of and against the null and alternative hypotheses. Likewise, although many spaceflight studies find significance, they are often constrained by difficulty in obtaining sufficient power. In a Bayesian framework, power constraints are less important, and it is possible to find evidence even with a small subject pool. Increasing the amount of evidence available reduces the width of the posteriors, increasing our confidence in the estimates, but even a small amount of data is better than no data. Finally, we believe that the Bayesian methodology provides an improved understanding of the dose–response parameters. Rather than an often‐misunderstood interpretation of a confidence interval, the Bayesian credible interval provides estimates regarding where the effect sizes lie within the population.

An additional benefit of a Bayesian workflow is that it provides the ability to construct more complicated models that are no longer constrained by the assumptions of (generalized) linear mixed‐effects models. Thus, rather than analysing each variable independently, we constructed a single, highly complex regression model to determine the multivariate structure of the response. The resulting output, in Figure 11, represents a novel understanding of the relationships between the physiological variables considered in the study.

We believe that this Bayesian workflow could be applied to spaceflight studies on physiological responses outside of the cardiovascular system. In particular, it is theoretically possible to elicit the relationship between multiple different physiological systems. For example, this framework will provide insight into questions such as, ‘What is the relationship between cardiovascular degradation and musculoskeletal remodelling during long‐duration spaceflight?’. Even with the low number of subjects typically included in spaceflight studies, evidence can be built up over time by replacing priors with posteriors from previous studies.

### 4.3 Limitations

There are two key limitations to this study: (1) we consider only the acute response to LBNP; and (2) we are limited to non‐invasive measurements. Regarding the acute nature of the study, Lightfoot et al. (1989) found that adaptation occurs with presyncopal symptom‐limited LBNP (over the course of a 9 day repeated exposure, with LBNP tolerance increasing by 47% over the first five exposures. The authors note an increase in RPP and maximal HR by days 7 and 8, but no change in the MAP response. Lightfoot et al. (1989) do not comment on adaptation to LBNP when exposed to less than presyncopal strength, but their work notes that adaptation is possible. This indicates that subjects with a low tolerance to LBNP, such as some of our female subjects, might be able to adapt to −50 mmHg LBNP. However, in multiple other studies, LBNP tolerance was found to be highly repeatable in any given individual (Convertino, 2001; Goswami et al., 2009; Howden et al., 2001; Lightfoot et al., 1991). When considering LBNP application prior to presyncope, multiple studies by Convertino and Goswami have confirmed that individual cardiovascular response is highly reproducible, even with rest periods as long as 1 year between tests (Convertino, 2001; Convertino et al., 2004; Goswami et al., 2019, 2008). This reproducibility also supported our decision to progress, rather than randomize, the presentation of LBNP strength to each subject. In order to determine the utility of LBNP as a spaceflight countermeasure to mitigate SANS or VTE, long‐term studies must be conducted, either in spaceflight or in HDT bed rest, to determine whether a periodic unloading of the cephalad fluid shift is able to prevent the manifestation of presyncopal symptoms.

Regarding the limitations of non‐invasive measurements, a direct measurement of CO could provide the most accurate dose–response relationship, because there are observable differences between the results of different methodologies (Geerts et al., 2011; Whittle et al., 2022). Furthermore, for the autonomic measures, samples of blood plasma catecholamines and other neurohormones along with intracellular magnesium levels (Benditt et al., 2020; Takase et al., 2004) could provide further insights into cardiovascular control. Finally, invasive measurement of central venous pressure could provide informative data on cardiac loading conditions and thoracic blood volume (Gaffney et al., 1985; Su et al., 2019). The VeinPress device used in the study was chosen for its heritage of use in previous spaceflight and parabolic flight investigations (Lee et al., 2020; Marshall‐Goebel et al., 2019; Martin et al., 2016), thus facilitating a direct comparison between studies. It is acknowledged that invasive measures, such as venous catheterization, might provide more accurate measurements of IJVP. However, non‐invasive measurements using ultrasonography are well established in a clinical environment (Aronen et al., 1994; Uthoff et al., 2011, 2010). The ability to obtain invasive measures could also allow for an accurate measure of ICP (Petersen et al., 2019). Future studies should attempt to obtain some measure of ICP, which might become easier with advancing technology in the area (Kristiansson et al., 2013).

Finally, regarding female subjects, we recorded but did not control the menstrual phase. Hart et al. (2011) have noted that sex hormones affect the activity of autonomic pathways on both the sympathetic and parasympathetic sides. Given the fluctuation of hormones across the menstrual cycle, it is envisaged that this could potentially add additional variation to the autonomic response of female subjects, perhaps helping to explain differences in tolerance. Future work should examine the effect of the menstrual phase on LBNP tolerability.

## 5 CONCLUSION

We subjected 24 male and female subjects to graded LBNP to investigate the acute changes in multiple haemodynamic parameters, autonomic indices and head/neck haemodynamics across the range from 0 to −50 mmHg LBNP in both 0° supine and 15° HDT positions. Our data revealed a linear dependence on pressure for all metrics considered except for IJVP (which presented a logistic dose–response), with varying effect sizes of response. Based on the experimental data collected, we conducted a Bayesian multivariate analysis to construct dose–response curves for all variables across the ranges considered. These dose–response curves demonstrated anthropometrically driven sex‐dependent changes in some metrics related to systemic haemodynamics and supported evidence from previous studies regarding different autonomic activations between men and women. For each variable considered, we calculated the relative effect size of a 15° HDT‐induced cephalad fluid shift and the LBNP required to counteract it. In particular, we demonstrated the potential for LBNP to reduce jugular venous flow stagnation and provided a logistic dose–response. Finally, we calculated the relationship structures between all the variables considered, in addition to subject characteristics, finding correlation structures between many groups of variables. These findings provide data to support development of spaceflight countermeasures to mitigate the risk of cardiovascular degradation, VTE events, and SANS (although the lack of effect of LBNP on OPP warrants further investigation).

## AUTHOR CONTRIBUTIONS

Richard S. Whittle and Ana Diaz‐Artiles contributed to the conception and design of the study. Material preparation, data collection, and analysis were performed by all authors. The first draft of the manuscript was written by Richard S. Whittle and commented on by Ana Diaz‐Artiles. All authors read and approved the final manuscript and agree to be accountable for all aspects of the work in ensuring that questions related to the accuracy or integrity of any part of the work are appropriately investigated and resolved. All persons designated as authors qualify for authorship, and all those who qualify for authorship are listed.

## CONFLICT OF INTEREST

None declared.

## Data Availability

The datasets analysed for this study are publicly available; a repository can be found on GitHub: https://github.com/BHP‐Lab/LBNP‐Dose‐Response
